# A Healthy Lifestyle Can Slow Immune System Aging and Reduce Age-Related Chronic Inflammation: A Narrative Review

**DOI:** 10.3390/ijms27125605

**Published:** 2026-06-21

**Authors:** Marta Cąkała-Jakimowicz, Anna Domaszewska-Szostek, Monika Puzianowska-Kuźnicka

**Affiliations:** 1 Department of Human Epigenetics, Mossakowski Medical Research Institute, Polish Academy of Sciences, 02-106 Warsaw, Poland; mcjakimowicz@imdik.pan.pl (M.C.-J.); adomaszewska@imdik.pan.pl (A.D.-S.); 2 Department of Lifestyle Medicine and Longevity, School of Public Health, Centre of Postgraduate Medical Education, 01-826 Warsaw, Poland

**Keywords:** inflammaging, immunosenescence, nutritional interventions, diet, physical activity, stress reduction

## Abstract

Age-related decline in immune system function is characterized by reduced numbers of naïve lymphocytes, the accumulation of senescent cells, impaired function of all immune cell types, and chronic low-grade inflammation (inflammaging). These alterations contribute to increased susceptibility to infections and malignancies, as well as to autoimmunity and other age-associated diseases. This article reviews current evidence on lifestyle interventions that may mitigate immune aging. Lifestyle-related strategies, including regular physical activity, nutritional interventions (e.g., different diets, caloric restriction, and other fasting-related approaches), stress reduction, and vaccination, are discussed as key modulators of immune function and systemic inflammation. Notably, vitamin D supplementation has been shown to reduce the incidence of autoimmune diseases by 22%. In comparison, caloric restriction has led to a decrease in CRP and TNF-α by 40% and 50%, respectively. Emerging complementary approaches, such as mind–body practices and controlled cold exposure, show promise, though current evidence remains limited and inconsistent. Therefore, integrated lifestyle strategies may slow aging-related immune decline and support healthy aging. However, longitudinal trials are required to define the optimal intervention parameters, population-specific thresholds, and the long-term durability of immune rejuvenation.

## 1. Introduction

Immune system aging is a cause of increased morbidity and mortality among older adults. Immunoaging involves the involution of lymphatic organs and senescence of immune cells (immunosenescence). This leads to a decline in naïve T and B cells as well as to attenuated function of natural killer (NK) cells, macrophages, neutrophils, and dendritic cells (DCs). The predominance of pro-inflammatory cytokines over anti-inflammatory cytokines leads to chronic, low-grade inflammation (inflammaging) [1,2]. Immunoaging combined with unfavorable environmental influences and an inappropriate lifestyle (environmental pollution, poor dietary habits, lack of physical activity, occupational stress, etc.) is associated with the development and progression of age-related diseases [3]. The process of cellular aging is associated with the accumulation of DNA damage, telomere shortening, epigenetic drift, loss of proteostasis, mitochondrial dysfunction, and impaired intercellular communication [4], leading to persistent cell cycle arrest, morphological, metabolic, and functional abnormalities, and relative resistance to apoptosis [5,6]. Senescent cells are also characterized by increased activity of lysosomal senescence-associated β-galactosidase (SA-β-Gal), nucleolus enlargement, chromatin rearrangement, nuclear lamina breakdown and persistent expression of DNA damage markers such as phosphorylated histone H2AX (γH2AX) and p53-binding protein 1 (53BP1) [7]. In addition, senescent cells secrete interleukins (IL)-1, -6, -8, -13, and -18; tumor necrosis factor (TNF) and its receptors; chemokines GROα and β, C-C motif chemokine ligand (CCL)-2, -5, -16, -26, and -20; growth factors HGF, FGF, TGF-β, and GM-CSF; matrix metalloproteases (MMP)-1, -10, and -3; the tissue plasminogen activator (tPA) and urokinase plasminogen activator (uPA); tissue inhibitors of metalloproteases (TIMP); the plasminogen activator inhibitor (PAI) and insulin-like growth factor binding proteins (IGFBP); various miRNAs, and other pro-inflammatory signals such as reactive oxygen and nitrogen species (ROS and RNS, respectively), collectively termed the senescence-associated secretory phenotype (SASP) that exacerbates age-related chronic inflammation [8,9,10,11,12]. The composition of SASP varies depending on the cell type, tissue environment, and the primary aging stimulus [10]. Studies have shown that immunosenescence is associated with chronic antigenic stress caused by latent, chronic infections, particularly with cytomegalovirus (CMV), herpes viruses, hepatitis B virus (HBV), human papillomavirus (HPV), or human immunodeficiency virus (HIV), and the need to maintain immune surveillance of these infections [13,14].

The molecular pathways involved in immunosenescence and inflammaging include the nuclear factor kappa-light-chain-enhancer of activated B cells (NF-κB) and NOD-, LRR- and pyrin domain-containing protein 3 (NLRP3) inflammasome signaling, mitochondrial dysfunction, decreased AMP-activated protein kinase (AMPK) activity (mTOR/AMPK axis), upregulation of the PI3K/Akt/mTOR pathway, and regulation of autophagy and SASP, all interconnected and mutually reinforcing. The dysfunction of one component exacerbates damage to the others, creating a self-perpetuating cycle.

Immunosenescence and inflammaging are closely linked to the continuous activation of the transcription factor NF-κB [15]. In the aging organism, the signal for the induction of this pathway comes from two main sources: external membrane receptors and internal cellular damage [16]. Classical activation occurs through elevated levels of circulating cytokines, such as TNF-α and IL-1β, which stimulate cells through a positive feedback loop. Additionally, weakened autophagy and leaky tissue barriers expose cells to pathogen-associated molecular patterns (PAMPs) and damage-associated molecular patterns (DAMPs), which, by primarily signaling through toll-like receptors 2/4 (TLR2/4), activate NF-κB and drive SASP production [15,16]. In addition to these classic membrane pathways, mitochondrial dysfunction, which progresses with age and generates destructive signals from within the cell, becomes a key initiator of inflammation. As a result of mitochondrial membrane damage, mitochondrial DNA (mtDNA) and mitochondrial RNA (mtRNA) leak into the cytosol. The released genetic material intensifies inflammation through the cytoplasmic cGAS/STING (DNA-specific) and RIG-I/MDA5/MAVS (RNA-specific) receptor pathways [17]. Both mitochondrial and nuclear signals influence NF-κB activation, which drives the transcription of pro-inflammatory genes [18]. Cytosolic material, including leaked mtDNA, extracellular adenosine triphosphate (ATP), ROS, and protein aggregates, also activates the NLRP3 inflammasome. NLRP3 activation triggers caspase-1 activation, leading to the maturation of the pro-inflammatory IL-1β and IL-18 cytokine precursors, which, in turn, are associated with several age-related diseases, such as metabolic syndrome. In this environment, ROS act as intracellular signaling molecules that enhance NF-κB activity. Instead of undergoing apoptosis, the cell enters cell cycle arrest and begins to secrete a range of senescence-promoting factors into the microenvironment [16]. Chronic SASP secretion and elevated NF-κB levels affect immune cells, including T and B lymphocytes, macrophages, and NK cells, leading to immunosenescence. Senescent cells accumulate in the body, continuously producing SASP, leading to a systemic, low-grade inflammation. Inflammatory cytokines circulating in peripheral blood and tissues bind to membrane receptors on healthy cells, reactivating the NF-κB signaling pathway, inducing excessive ROS production, and damaging healthy mitochondria, thereby fueling aging and the development of age-related diseases [19,20].

Under physiological conditions, damaged organelles and toxic protein aggregates are continuously removed through mitophagy (selective autophagy of damaged mitochondria), autophagy, and the ubiquitin–proteasome system. However, in aging cells, this process is impaired. With age, AMPK activity decreases, which, under homeostatic conditions, stimulates mitophagy and directly silences the NLRP3/NF-κB inflammatory pathways. This decline leads to hyperactivation of the mechanistic target of rapamycin (mTOR) pathway—an intracellular signaling cascade that regulates cell growth, proliferation, metabolism, and survival. Dysregulation of this pathway also plays a significant role in carcinogenesis [21]. Chronic activation of the mTOR pathway inhibits autophagy and mitophagy. This allows damaged mitochondria to accumulate, generating more ROS, leaking more mtDNA and mtRNA into the cytosol, and thereby amplifying cGAS/STING and RIG-I/MDA5/MAVS-dependent signaling and continuously reactivating NF-κB and the NLRP3 inflammasome, which closes the vicious self-reinforcing cycle of inflammaging [22].

Many lifestyle interventions can inhibit NF-κB through diverse molecular mechanisms. Regular physical exercise effectively counteracts chronic inflammation by modifying the DNA methylation profile of immune cells and promoting their anti-inflammatory phenotype [23]. Mindfulness meditation reduces the expression of key histone deacetylase genes, leading to changes in inflammatory gene transcription that persist beyond the practice session itself [24]. Omega-3 fatty acids and flavonoids act at the level of lipid mediator signaling [25,26], while vitamin D acts through the VDR nuclear receptor [27]. Stress reduction techniques such as mindfulness-based stress reduction (MBSR) and cognitive-behavioral stress management (CBSM) reduce inflammation by regulating the hypothalamic–pituitary–adrenal (HPA) axis [28,29]. A calorie-restricted diet (CR) suppresses NLRP3 inflammasome activation by reducing the platelet-activating factor acetyl hydrolase (*PLA2G7*)-encoding gene expression [30], as well as through combined resistance-aerobic training, which stimulates kynurenic acid production [31]. Quercetin is present in a range of plant foods and has been documented to have NLRP3-inhibitory properties [32]. Reducing NLRP3 inflammasome activation may protect against age-induced thymic lipoatrophy, thereby supporting T-cell production [30]. Furthermore, the Dietary Approaches to Stop Hypertension (DASH) diet is associated with a significant reduction in the oxidative stress biomarker malondialdehyde (MDA) [33]. The Mediterranean-DASH Intervention for Neurodegenerative Delay (MIND) diet reduces intracellular ROS production [34], and vitamin C directly neutralizes ROS [35]. An effective, non-pharmacological method for maintaining mitochondrial function and optimizing the lipid profile of mitochondrial membranes involves a diet rich in ω-3 fatty acids and monounsaturated fatty acids (MUFA) from extra virgin olive oil [36,37]. In turn, calorie restriction effectively reduces mTOR pathway activity, thereby increasing autophagy in immune cells and facilitating the removal of intracellular pathogens [38]. Furthermore, some flavonoids, by inhibiting the PI3K/Akt/mTOR axis, induce apoptosis in cancer cells [39] and promote senolysis, reducing the body’s SASP burden [32].

In this article, we describe in more detail natural therapies and lifestyle interventions that support the immune system by reducing inflammation and oxidative stress, thereby improving quality of life, slowing the progression of aging, and, consequently, increasing the likelihood of successful aging.

## 2. Methodology

This narrative review presents the current state of knowledge regarding the impact of a healthy lifestyle on immunosenescence and inflammaging. This study considers dietary interventions, caloric restriction, physical activity, sleep, and psychological well-being as factors influencing these processes. A comprehensive literature search was conducted on PubMed, Scopus, and Web of Science using search terms related to immunosenescence, inflammation, dietary interventions, caloric restriction, physical activity, stress reduction, cold exposure, vaccination, immune aging, and combinations thereof. This review emphasizes randomized controlled trials (RCTs) in humans, prospective cohort studies, and cross-sectional studies; where human data were limited, animal and in vitro studies were included. The utilization of animal models proved instrumental in elucidating molecular mechanisms, while human data remained mostly observational.

## 3. Diet

### 3.1. Nutrients and Bioactive Compounds

External factors modulate the aging process [40,41]. Among them, a well-balanced diet is essential for the proper functioning of the immune system and is particularly important for older adults. By supplementing deficiencies to recommended levels, resistance to infections can be increased and lifespan extended [42]. Therefore, increasing attention is being paid to dietary patterns and specific nutrients that can modulate inflammatory processes, lower blood pressure, improve cognitive function, and thus promote healthy aging and longevity (Table 1 and Table 2). To implement an anti-inflammatory diet, it is recommended to replace refined grains with whole grains, saturated and trans fats with healthy plant-based unsaturated fats, and increase consumption of colorful vegetables and fruits, while limiting sugary and highly processed foods. Furthermore, it is recommended to choose protein sources such as fish and legumes instead of red and processed meats; use herbs and spices like turmeric, ginger, garlic, and cinnamon instead of salt and sugar; and consume probiotic-rich foods such as yogurt and kefir instead of processed and sugary snacks. Ample evidence indicates that spices and herbs have antioxidant, anti-inflammatory, anticancer, and glucose- and cholesterol-lowering properties. They owe their health benefits to bioactive components, including sulfur-containing compounds, tannins, alkaloids, phenolic diterpenes, vitamins, and especially flavonoids and polyphenols. Spices and herbs such as cloves, rosemary, sage, oregano, and cinnamon are excellent sources of antioxidants due to their high levels of phenolic compounds [43]. Other foods with anti-inflammatory, antioxidant, antiviral, and immune-boosting properties include turmeric, ginger, garlic, onion, and saffron [44]. Foods rich in dietary fiber (prebiotics), vitamins C, D, and E, ω-3 fatty acids, and zinc are also important for maintaining health [45,46].

In aging rats, age-related changes in lymph nodes were slowed following the administration of a bioactive phytonutrient composition containing plant flavonoids and trace elements, including zinc, selenium, iron, manganese, and copper. Improved lymph flow was also demonstrated [47]. Therefore, older individuals are advised to supplement their diet with a combination of micronutrients, especially those that play a key role in the immune system, such as plant polyphenols, vitamins C, D, and E, zinc, selenium, docosahexaenoic acid (DHA), and eicosapentaenoic acid (EPA) [48]. Note that the effectiveness of an anti-inflammatory diet results not from the presence of individual components but from their comprehensive, synergistic, mutually reinforcing effects [46].

#### 3.1.1. Vitamins C and E

Vitamin C (L-ascorbic acid) is an important component of an anti-inflammatory diet. Foods rich in vitamin C include tomatoes, potatoes, green leafy vegetables, berries, and citrus fruits. It is a powerful antioxidant that neutralizes ROS, protecting proteins and DNA from oxidative stress and damage [49]. Vitamin C plays a key role in innate and adaptive immunity. It accumulates in phagocytic cells and can enhance chemotaxis, phagocytosis, and microbial clearance. It is also essential for apoptosis and the removal of worn-out neutrophils by macrophages at sites of infection, thereby reducing excessive neutrophil extracellular trap (NET) formation and necrosis. Furthermore, it has been shown to support the differentiation and proliferation of B and T lymphocytes [35]. Vitamin C inhibits the growth of many pathogens, such as *Pseudomonas aeruginosa*, *Staphylococcus aureus*, and *Escherichia coli*; hinders the replication of viruses such as herpes simplex and rabies viruses; and has antiparasitic activity against *Trypanosoma cruzi* and *Plasmodium* infections. Furthermore, vitamin C can inhibit the morphogenesis of some fungi, such as *Candida albicans* [50,51]. Mice fed a high dose of vitamin C for 1 year had higher thymus mass, as well as total leukocyte, lymphocyte, granulocyte, and monocyte counts in peripheral blood, as well as splenocyte and thymocyte counts compared to control mice. The number of naïve T cells in peripheral blood and clusters of differentiated CD4^+^CD8^+^, CD4^+^CD8^−^, or CD4^−^CD8^+^ T cells among thymocytes were significantly higher in animals consuming vitamin C. The authors suggest that long-term high-dose vitamin C intake inhibits thymic involution associated with aging [52]. It has also been observed that supplementation with 1 g of ascorbic acid and 400 mg of vitamin E for 28 days significantly increased IL-1β and TNF-α production in healthy adults. In vitro studies on peripheral blood cells have shown that vitamins C and E, when combined, reduced lipopolysaccharide (LPS)-induced prostaglandin E2 (PGE-2) production and increased TNF-α production. These results demonstrate the potent immunopotential of combined vitamin C and E supplementation in adults [53]. Dendritic cells treated with vitamins C and E were resistant to phenotypic and functional changes following stimulation with pro-inflammatory cytokines. Following treatment, intracellular free radical levels decreased, preventing the activation of key pathways such as NF-κB, p38 MAPK, and protein kinase C, which are involved in the inflammatory response. Allogeneic T cells were anergized following exposure to vitamin-treated DCs and secreted higher levels of Th2 cytokines and IL-10 than cells incubated with control DCs. These data indicate that DCs treated with vitamins C and E may support the induction of tolerance to alloantigens or autoantigens and could therefore be used in therapies that induce immune tolerance, for example, to prevent transplant rejection or treat autoimmune diseases [54]. A 30-day vitamin E supplementation regimen improved the immune response in healthy elderly individuals to several skin-applied antigens (e. g., tetanus and diphtheria toxoid, streptococcus group C, mycobacterium tuberculosis, candida, tricophyton, and proteus) in a delayed-type hypersensitivity skin test (DTH) and increased IL-2 production, which enhances lymphocyte function, increases the mitogenic response to concavalin A, and reduces plasma lipid peroxide concentration and lymphocyte activity-inhibiting PGE2 synthesis by peripheral blood mononuclear cells (PBMC) [55]. Therefore, consistent intake of approximately 200 mg/day of vitamin C via diet and supplements seems crucial [35].

#### 3.1.2. Zinc

Zinc is another nutrient that supports the immune system and reduces inflammation in older adults. Sources of zinc include meat, fish, seafood, eggs, dairy products, beans, nuts, and whole grains. The bioavailability of zinc from plant foods is lower than from animal products [56]. Zinc increases neutrophil chemotaxis, promotes the cytotoxic activity of NK cells, supports the phagocytic function of macrophages, and preserves T- and B-cell responsiveness. The presence of zinc increases regulatory T-cell (Treg) activity and restores immune homeostasis by reducing inflammation, balancing the Th1/Th2 ratio, and maintaining normal cytokine levels and B cell function. The effects of zinc deficiency are similar to age-related immune dysfunction, leading to impaired adaptive immunity and an increased pro-inflammatory response. Zinc deficiency is associated with increased production of pro-inflammatory cytokines IL-1, IL-6, and TNF-α by antigen-presenting cells, an enhanced Th2 response, and a decreased Th1 response. These changes result in reduced IFN-γ and IL-2 production, reduced immunoglobulin class switching, nonspecific B-cell activation, and impaired Treg cell function, thus promoting inflammation and immune imbalance [57]. Eight weeks of 45 mg daily zinc supplementation in healthy adult volunteers reduced plasma levels of malondialdehyde (MDA) and 4-hydroxyalkenals (HAE) lipid peroxidation products, and 8-hydroxy-2′-deoxyguanoside (8-OHdG) biomarker of oxidative DNA damage compared to baseline values. Decreased NF-α and IL-1β mRNA levels following LPS stimulation were observed in mononuclear blood cells (MNCs) of individuals receiving zinc supplementation compared to placebo. Zinc had a protective effect against TNF-α-induced NF-κB activation. These data support the role of zinc in reducing oxidative stress and modulating inflammatory pathways, suggesting its potential to delay or reduce the progression of age-related diseases, including diabetes, cardiovascular disease, depression, and vision loss [58]. The Food and Nutrition Board at the National Academies of Sciences, Engineering, and Medicine has established recommended daily zinc intakes of 8 to 12 mg for adults and 2 to 13 mg for infants, children, and adolescents, depending on age, gender, and life stage [59].

#### 3.1.3. Omega-3 Fatty Acids and Monounsaturated Fatty Acids (MUFA)

Omega-3 fatty acids can directly suppress the inflammatory response and modulate metabolic processes; their benefits have been observed in animal models of rheumatoid arthritis (RA), inflammatory bowel disease (IBD), and asthma [25]. Foods rich in ω-3 fatty acids, including fatty fish, flaxseed, chia seeds, walnuts, and fish oil supplements, have been shown to inhibit leukocyte chemotaxis, reduce the expression of adhesion proteins, and reduce interactions between leukocytes and vascular endothelial cells. They also reduce the production of eicosanoids such as prostaglandin E2 (PGE2), thromboxane B2 (TXB2), leukotriene B4 (LTB4), hydroxyeicosatetraenoic and epoxyeicosatrienoic acids, and leukotriene E4 (LTE4) due to inflammatory cells from arachidonic acid, the production of pro-inflammatory cytokines, and the reactivity of T lymphocytes [60]. At the same time, eicosapentaenoic acid is a precursor of eicosanoids such as prostaglandin E3 (PGE3), thromboxane (TXA3), leukotriene B5 (LTB5), and leukotriene E5 (LTE5), which often have lower biological activity than those produced from arachidonic acid. EPA and DHA contribute to the formation of resolvins and protectins, which have anti-inflammatory properties [60,61]. EPA competes with arachidonic acid for key enzymes, cyclooxygenase (COX) and lipoxygenase (LOX), producing eicosanoids with lower inflammatory potential than arachidonic acid derivatives. As a result, the EPA-to-archidonic acid ratio may serve as a marker of chronic inflammation, with lower values indicating greater inflammation [62]. The mechanisms underlying the anti-inflammatory effects of ω-3 fatty acids include altering the composition of membrane phospholipids, influencing lipid raft formation at the cell membrane, and inhibiting NF-κB activation, as described, for example, in T lymphocytes [63]. There appear to be at least two mechanisms by which ω-3 polyunsaturated fatty acids inhibit NF-κB activation: one involving GPR120 and the other involving NR1C3 [25]. The recommended daily intake of EPA and DHA depends on age and health status. It ranges from 40 to 250 mg in children and adolescents and from 200 mg to 600 mg in adults [64]. Monounsaturated fatty acids (MUFAs) present in olive oil, avocado, nuts, seeds, and rapeseed oil increase adiponectin secretion, improve insulin sensitivity, and reduce the number of pro-inflammatory cytokines and circulating monocytes [65].

#### 3.1.4. Dietary Fiber

Dietary fiber is found in whole grains, fruits, vegetables, legumes, nuts, and seeds. It increases the production of anti-inflammatory short-chain fatty acids (SCFAs) such as butyrate, propionate, and acetate in the large intestine, helps maintain a healthy gut microbiome, and reduces the amount of pro-inflammatory cytokines in the bloodstream [66]. Other nutrients, such as probiotics and ω-3 fatty acids, may also increase gut microbiome diversity and reduce inflammation. Lactic acid bacteria and bifidobacteria prevent intestinal hyperpermeability and reduce LPS-dependent chronic inflammation. Moreover, ω-3 fatty acids increase the number of beneficial *Bifidobacteriae*, which inhibit the action of LPS, and reduce the number of unfavorable *Enterobacteriae* that produce LPS. The combination of probiotics with ω-3 fatty acids is a promising strategy for regulating immunity by influencing the intestinal microbiota, with potentially beneficial effects on inflammation, which is common in the elderly and obese [67].

#### 3.1.5. Polyphenols and Flavonoids

Polyphenolic compounds of plant origin, most notably flavonoids, deserve particular attention because they can modulate the immune system. Antioxidants and flavonoids present in fruits such as berries and citrus, vegetables such as spinach and kale, nuts, seeds, and herbs neutralize free radicals, alleviate oxidative stress, and protect against DNA damage [68]. Treatment with the citrus flavonoid nobiletin activates the IL-6/STAT3/FOXO3a signaling pathway in macrophages by reducing IL-6 and STAT3 phosphorylation and increasing FOXO3a phosphorylation in the cell nucleus. This pathway induces macrophage autophagy, an important mechanism for regulating inflammation [69]. Dual COX/5-LOX inhibitors are promising new anti-inflammatory drugs. Curcumin, capsaicin, and gingerol inhibit the synthesis of pro-inflammatory prostaglandins and leukotrienes by targeting COX and LOX [70]. Flavocoxid, a mixture of plant-derived flavonoids baicalin and catechin, also inhibits COX1/2 and 5-LOX and is effective in inhibiting NF-κB-induced production of pro-inflammatory cytokines. Mice modeling Alzheimer’s disease treated with flavocoxid showed improved cognitive function; reduced eicosanoid production; reduced phosphorylation of amyloid precursor protein (APP-pThr668), Aβ_1-42_, p-tau (pThr181), and pERK; and reduced NLRP3 inflammasome activation, Aβ plaque formation, and neuronal loss compared to animals treated with saline [26]. Dual COX/5-LOX pathway inhibitors based on flavonoids exhibit less gastrointestinal toxicity, fewer cardiovascular side effects, and broader anti-inflammatory effects than traditional nonsteroidal anti-inflammatory drugs. Inhibition of the production of both prostaglandins and leukotrienes, which are inflammatory mediators of arachidonic acid metabolism, may have a synergistic effect, resulting in significant suppression of inflammatory reactions [71,72].

Flavonoids have demonstrated significant immunomodulatory effects on innate and adaptive immune cells by regulating receptor and costimulatory molecule expression, inflammatory factor secretion, and signaling pathway function. They act not only as antioxidants but also as intelligent regulators of the immune system. They demonstrate the ability to simultaneously suppress excessive inflammatory responses (autoimmunity/allergies) and enhance defense mechanisms against cancer and infections [73]. Naringenin, chrysin, and hesperetin increase NK cell proliferation, viability, activation, and cytotoxicity [74,75,76]. Apigenin, quercetin, chrysin, and epigallocatechin-3-gallate (EGCG) inhibit the maturation and differentiation of dendritic cells, which may prevent autoimmune diseases because immature dendritic cells promote immune tolerance rather than attack their own tissues [77,78,79,80,81]. Quercetin, luteolin, and mairin inhibit the activation of M1 macrophages and promote their conversion to M2 [82,83,84]. EGCG plays an immunomodulatory role by counteracting neutrophil-mediated damage in the context of persistent or uncontrolled inflammation [85]. Flavonoids also regulate the adaptive immune response by supporting the proliferation of Treg lymphocytes and increasing the activity of cytotoxic T lymphocytes (CTLs), while inhibiting the activation or proliferation of Th (T helper) lymphocytes. Flavonoids may be part of a potential treatment plan for patients with a hyperactive immune system or immunodeficiency [39,73,75].

Chronic inflammation is a factor contributing to the development of human cancers and is involved in all stages of carcinogenesis [86]. Moreover, tumors create an immunosuppressive microenvironment that prevents the immune system from effectively fighting them. The flavonoid icaritin is currently undergoing Phase 3 clinical trials for the treatment of advanced hepatocellular carcinoma. Icaritin enhances the activation and proliferation of CD8^+^ T lymphocytes and increases their ability to recognize tumor antigens presented by major histocompatibility complex (MHC) class I molecules. Furthermore, it increases the production of pro-inflammatory cytokines (e.g., IFN-γ) and chemokines, which attract additional immune cells to the tumor, enhancing their cytotoxic activity and leading to more effective destruction of tumor cells. Icaritin reduces the number and accumulation of myeloid-derived suppressor cells in the tumor microenvironment and inhibits their immunosuppressive functions, including reduced expression of enzymes such as arginase 1 (ARG1) and nitric oxide synthase (iNOS), which deactivate T lymphocytes. It may also induce their differentiation toward more mature, less suppressive cells, such as dendritic cells. It reprograms anti-inflammatory M2 macrophages, which promote tumor progression, to the M1 phenotype. M1 macrophages more effectively phagocytize tumor cells and secrete pro-inflammatory cytokines, thereby enhancing the immune response. Icaritin’s effects on neutrophils include reducing their ability to form NETs and increasing their bactericidal activity and tumor cell-killing capacity [87]. Some flavonoids have been shown to inhibit the PI3K/Akt/mTOR axis and induce apoptosis in cancer cells. These include fisetin, curcumin, quercetin, baicalin, and butein [39].

#### 3.1.6. Vitamin D

Vitamin D is synthesized in the skin and hydroxylated to its active form in the liver and kidneys. In older adults, endogenous synthesis is often insufficient, requiring dietary consumption and dedicated supplements. Vitamin D is present in fatty fish, beef liver, cheese, egg yolk, mushrooms, and some vegetables [88,89]. Most immune system cells possess the vitamin D nuclear receptor (VDR) [89,90,91]. Antigen-presenting cells, such as macrophages and dendritic cells, also possess 25-hydroxyvitamin D 1α-hydroxylase (CYP27B1) and can actively metabolize 25-hydroxyvitamin D to active 1α,25-dihydroxyvitamin D (1,25(OH)_2_D) [92]. CYP27B1 expression is induced by pattern recognition receptors (PRRs) found on the surface of immune cells. This leads to local production of 1,25(OH)_2_D at the site of infection, which, in turn, induces the expression of genes encoding antimicrobial peptides, such as cathelicidin antimicrobial peptide/LL-37 (CAMP), defensins, hepcidin and neutrophil peptides, which act as endogenous antibiotics against various pathogens [93,94]. Vitamin D enhances the cytotoxic activity of NK cells against infected cells and the recruitment of neutrophils [90,95]. T and B lymphocytes express significant amounts of VDR and CYP27B1 after activation [96,97]. Furthermore, 1,25(OH)_2_D and VDR directly regulate NF-κB activation, a pathway essential for the inflammatory response [27]. Conversely, 1,25(OH)_2_D controls inflammation by delaying dendritic cell differentiation, inducing a tolerogenic phenotype, inhibiting antigen presentation, and limiting T-cell activation and proliferation. Reducing the expression of the pro-inflammatory Th17 cell subset, increasing Treg expression, and increasing anti-inflammatory cytokine concentrations, such as IL-4, IL-5, IL-10, and CCL2, may alleviate inflammation and autoimmune diseases. 1,25(OH)_2_D inhibits the proliferation and immunoglobulin production of B lymphocytes [90,98,99,100,101,102], and 1,25(OH)_2_D stimulates autophagy, which is a critical mechanism for controlling intracellular pathogens such as *Mycobacterium tuberculosis* [102,103]. Artusa et al. observed that 1,25(OH)_2_D via VDR increases the expression of autoimmune regulator (AIRE) in thymic stromal cells. This indicates that 1,25(OH)2D signaling is involved in the establishment of central tolerance, which underlies the link between vitamin D deficiency and increased autoimmunity [104]. Vitamin D supports mitochondrial autophagy, which prevents excessive ROS production; reduces the expression of inflammatory cytokines such as TNF-α; and prevents oxidative stress. It acts synergistically with KLOTHO and NRF2 proteins, increasing antioxidant levels [105]. Analysis of the association of vitamin D deficiency with aging-related immune dysfunction in people over 60 years of age has shown that IL-6, CRP, and IL-6/IL-10 and CRP/IL-10 ratios were significantly higher in individuals with serum 25(OH)D deficiency (<25 nmol/l) compared to those with sufficient levels (>75 nmol/l) after adjustment for age, sex, and body mass index [106]. A study involving 25,871 participants assessed the effect of daily intake of 2000 IU vitamin D, 1000 mg of ω3 fatty acids, or placebo on the incidence of autoimmune diseases [107]. Vitamin D supplementation, with or without ω3 fatty acids, reduced the incidence of all self-reported autoimmune diseases by 22%. Two years after the study ended, the protective effect of vitamin D disappeared [108].

Based on abundant data on the impact of nutrients on body function, including immune system function, and in the context of aging, scientific and medical societies and research groups have developed diets that ensure optimal nutrition. These diets share a common characteristic: they recommend consuming large amounts of vegetables, fruits, nuts, seeds, and minimally processed foods. Examples of such diets are briefly described below.

### 3.2. Dietary Patterns

The following dietary patterns have the strongest evidence base for immune and anti-inflammatory outcomes in aging populations.

The **Mediterranean diet (MD)** has been shown to extend the healthy phase of life, by influencing immune system function, reducing inflammation, and lowering ROS levels. Many MD variants exist, but all are characterized by a high consumption of plant products, including vegetables, fruits, legumes, whole-grain bread, cereals, nuts, seeds, and olive oil. Consumption of dairy products, fish, poultry, and wine is moderate, while that of red meat and sweets is low. The Mediterranean diet is rich in vitamins, minerals, polyphenols, fiber, nitrates, polyunsaturated fatty acids (PUFAs), and MUFAs [109,110,111]. Among ω-6 polyunsaturated fatty acids, linoleic acid is essential, while among ω-3 polyunsaturated fatty acids, EPA and DHA are essential. They are found mainly in fish oils [112]. Antioxidants and other bioactive compounds found in the MD, such as melatonin, phytoestrogens, carotenoids, and polyphenols, may prevent oxidative damage and inflammation, promote DNA repair, and slow telomere shortening [113,114,115,116]. Olive oil components, such as oleocanthal and oleuropein, also have antioxidant and anti-inflammatory properties [117,118,119]. Indeed, the MD enriched with additional extra virgin olive oil used by patients with high cardiovascular risk has yielded anti-inflammatory effects by reducing CRP, IL-6, and TNFα levels [120,121,122]. Similarly, 2 years of MD use by patients with metabolic syndrome led to reduced serum concentrations of CRP, IL-6, IL-7, and IL-18, reduced insulin resistance, and improved vascular endothelial [123]. Furthermore, ω-3 fatty acids and red wine help maintain normal mitochondrial function, the dysfunction of which is associated with oxidative damage [36]. MUFAs from olive oil help optimize the lipid profile of mitochondrial membranes, increasing their resistance to oxidative damage [37]. By reducing ROS concentration, MD reduces DNA damage and the number of senescent cells [124,125]. Mechanistically, it reduces the production of pro-inflammatory mediators, e.g., resveratrol inhibits NF-κB transcription, which is essential for SASP genesis [126,127]. Dietary components such as quercetin, by inhibiting the PI3K-AKT pathway, may facilitate the programmed death of senescent cells, preventing their harmful accumulation in the body [32]. The MD reduces plasma concentrations of sICAM-1, sVCAM-1, sP-selectin, IL-6, and CRP in patients at high cardiovascular risk. Blood vessels become more resistant to inflammatory cell adhesion, which directly inhibits the development of atherosclerosis [128,129,130]. A 1-year MD enriched with extra virgin olive oil and/or nuts reduced the expression of adhesion molecules on T lymphocytes (CD49d, CD40) and monocytes (CD49d, CD40) in individuals at high risk of cardiovascular disease compared to the low-fat diet group, which reduced the likelihood of new inflammatory cell influx into the vessel wall. Similarly, parameters directly related to atherosclerotic plaque stability, such as IL-18 and the IL-18/IL-10 ratio, showed a favorable profile [128,130]. MD enriched with an additional dose of extra virgin olive oil or nuts has reduced the risk of cardiovascular disease, improved classic cardiovascular risk factors, and exerted anti-inflammatory effects. Its effects on lipids and blood pressure were evident after 3 months, and the maximum effect on systemic inflammatory biomarkers was observed after 1 year [128]. A study on 7447 individuals showed that an MD rich in olive oil or nuts reduced the risk of cardiovascular disease by 30% compared with a low-fat diet [131]. Koelman et al., in a systematic review and meta-analysis of randomized controlled trials, identified MD as the dietary pattern with the strongest anti-inflammatory potential and a safe, long-term use profile. The synergy and balanced combination of all MD components undoubtedly possess strong anti-inflammatory and immune-balancing properties, justifying its recommendation for maintaining strong immune health [132].

The **Dietary Approaches to Stop Hypertension (DASH) diet** is an intervention that helps lower systolic blood pressure by approximately 6–11 mm Hg. It promotes the consumption of vegetables and fruits with a low glycemic index. Kale, broccoli, spinach, mustard greens, legumes, beans, bananas, oranges, whole grains, nuts, hemp seeds, and flax seeds are highly recommended. Consuming fats from olive oil, avocados, and fish is important to prevent inflammation. The DASH diet recommends limiting sodium to approximately 1500 mg/day and consuming minimally processed and fresh foods. The DASH diet also promotes foods rich in potassium, calcium, and magnesium [133,134,135]. Numerous studies have shown that this diet reduces all-cause mortality [136]. DASH may be a very useful tool for physicians, enabling more effective treatment of cardiometabolic diseases, including age-related ones, especially when combined with pharmacological intervention [135]. A meta-analysis of eight studies involving 317 participants showed that adherence to the DASH diet was associated with a significant decrease in malondialdehyde, a biomarker of oxidative stress, and a significant increase in glutathione (GSH), the body’s main antioxidant. The decrease in MDA levels indicates that this dietary model effectively protects cells from intracellular oxidation, an effect attributed to its high levels of polyphenols, vitamin C, magnesium, and potassium, as well as its low glycemic index. Furthermore, in the above study, an insignificant trend towards increased levels of total antioxidant capacity, nitric oxide (NO), and F2-isoprostanes was observed [33].

The **Mediterranean-DASH Intervention for Neurodegenerative Delay (MIND) diet** was developed in the USA as a combination of two established dietary models, the MD and DASH diet [137]. Its primary goal is to protect the brain and delay cognitive decline. Its key difference from baseline diets is its emphasis on foods with proven neuroprotective effects and the restriction of foods high in saturated fat [138]. The MIND diet’s effectiveness stems from its richness in antioxidant and anti-inflammatory nutrients, such as B vitamins, vitamin E, flavonoids, folic acid, carotenoids, and PUFAs. B vitamins, especially folic acid and vitamin B_12_, act as coenzymes that convert homocysteine to methionine. High homocysteine levels, a marker of inflammation used in medical practice, are a risk factor for cardiovascular disease and dementia. Carotenoids and polyphenols are powerful antioxidants found in abundance in green leafy vegetables and berries, neutralizing oxidative stress and inflammation. Carotenoids interact with various types of cell membranes and may also act indirectly through interactions with Nrf2, NF-κB, and MAPK protein kinase. They are absorbed by micelles and then activated by β-carotenoid oxygenases 1 and 2 to remove ROS. Resveratrol disrupts the amyloid cascade by reducing Aβ-induced ROS production, as well as tau protein phosphorylation and accumulation. Furthermore, anthocyanins and catechins, which cross the blood–brain barrier, protect neurons from oxidative stress-induced damage and inflammation [34]. PUFAs support the structure and function of nerve cells. DHA, EPA, and ω-3 fatty acids are the main components of membrane phospholipids, influencing membrane fluidity and signal transduction. α-linolenic acid has a strong anti-inflammatory effect, reducing iNOS, COX-2, and the inflammatory cytokines IL-1β, IL-6, and TNF-α, as well as Aβ fibril formation [139]. Studies have shown that DHA and EPA supplementation primarily leads to improved memory [140,141,142]. A study conducted on 3777 participants with a 7-year follow-up showed that adherence to the MIND diet was associated with a favorable profile of circulating inflammatory biomarkers, including lower levels of IL-1, IL-6, IL-10, sTNFR-1, C-reactive protein, neutrophils, leukocyte count, and cystatin C and higher albumin levels [143].

Long-term association between dietary patterns and indices and healthy aging, with regard to cognitive, physical, and mental health was analyzed: the Alternative Healthy Eating Index (AHEI), the Alternative MEDiterranean index (aMED), the DASH, the MIND, the Healthy Plant-based Diet (hPDI), the Planetary Health Diet Index (PHDI), the Empirically Inflammatory Dietary Pattern (EDIP), the Empirical Dietary Index for Hyperinsulinemia (EDIH), and ultraprocessed food (UPF). Adherence to all healthy patterns and the avoidance of pro-inflammatory and ultraprocessed foods correlated with healthy aging, with the AHEI demonstrating the strongest association. Preferential consumption of vegetables, whole-grain cereals, fruits, nuts, legumes, and unsaturated fats of plant origin, and moderate consumption of low-fat dairy products were associated with increased odds of healthy aging. Additional intake of unsaturated fats, including polyunsaturated fatty acids, was particularly beneficial. In turn, high consumption of trans fats, sodium, sweetened beverages, red or processed meats, and highly processed foods of all kinds was inversely correlated with healthy aging [144]. Consistent with this observation, Pang et al. demonstrated in mice that a long-term high-fat diet accelerates bone marrow aging and enhances adipogenesis in the bone marrow microenvironment. Myeloid macrophages expressed increased expression of chitinase-3-like protein 1 (Chil3) and fatty acid binding protein 4 (Fabp4), which are associated with the inflammatory response and adipocyte regulation. The Ptn–Sdc3 and Cxcl12–Cxcr4 axes between bone marrow macrophages and brain epithelial cells have also been discovered, described, and associated with the translocation of inflammation from the bone marrow to the brain of mice [145].

**Table 2:** Selected nutritional intervention studies demonstrating effects on inflammaging markers. *8-OHdG: 8-hydroxy-2′-deoxyguanosine; bw: body weight; CRP: C-reactive protein; DCs: dendritic cells; DTH: delayed-type hypersensitivity; EVOO: extra virgin olive oil; IL: interleukin; LPS: lipopolysaccharide; MDA: malondialdehyde; NF-κB: nuclear factor kappa-light-chain-enhancer of activated B cells; PGE2: prostaglandin E2; PREDIMED: Prevención con Dieta Mediterránea; RCT: randomized controlled trial; SASP: senescence-associated secretory phenotype; SMP-30: senescence marker protein-30; TNF-α: tumor necrosis factor α; VITAL: VITamin D and OmegA-3 TriaL.*

Dietary Intervention	Type of Study	Sample Size	Target	Inflammaging Markers	Effect of Intervention	Ref.
**Vitamin C** *high-dose, 200 mg/kg bw/day* vs. *20 mg/kg bw/day,* *1 year*	Animal RCT *(SMP-30 KO mice)*	N = 10 per group (standard vs. high dose VC)	Thymus, peripheral blood	Thymic mass, naϊve T cells, thymocyte counts	Higher thymus mass, more thymocytes and naϊve T cells, inhibition of age-related thymic involution	[52]
**Vitamin E** *800 mg/day,* *30 days*	Human RCT	N = 32 Healthy elderly (>60 years) men	Skin (DTH), peripheral blood	IL-2, PGE2, lymphocyte mitogenic response	Enhanced DTH responses, increased IL-2, reduced PGE2 synthesis	[55]
**Vitamins C + E** *1g + 400mg/d,* *28 days*	Human single-blind trial	N = 10 per group (VC vs. E vs. C + E) Healthy adults	Peripheral blood mononuclear cells	IL-1β, TNF-α, PGE2	Increased IL-1β and TNF-α, decreased PGE2, tolerogenic DCs phenotype	[53,54]
**Zinc** *45 mg/day,* *8 weeks*	Human RCT	N = 10 per group (zinc vs. placebo) Healthy adults (20–50 years)	Peripheral blood mononuclear cells	MDA, 8-OHdG, NF-κB, IL-1β, TNF-α	Reduced oxidative stress markers, reduced LPS-stimulated TNF-α and IL-1β, NF-κB protection	[58]
**Vitamin D** *2000 IU/day,* *5 years*	Human RCT (VITAL)	N = 25,871 Adults (≥50 years)	Systemic	Autoimmune disease incidence, IL-6, CRP	22% reduction in autoimmune disease with or without ω-3 fatty acids, protective	[107,108]
**Mediterranean diet + EVOO** *1 year*	Human RCT (PREDIMED)	N = 7447 Adults with high CV risk	Systemic/ vascular	IL-6, CRP, TNF-α, sICAM-1, sVCAM-1	~30% reduced risk of major cardiovascular events (HR 0.70, EVOO group); CRP, IL-6, IL-8, and TNF-α significantly reduced vs. low-fat diet in PREDIMED inflammatory biomarker sub-analyses	[131]
**MIND diet** *7-year follow-up*	Human prospective cohort	N = 3777 Adults	Systemic	IL-1, IL-6, IL-10, CRP, sTNFR-1, neutrophils	Adherence associated with lower IL-1, IL-6, CRP, neutrophil and leukocyte counts; and with higher albumin	[143]

### 3.3. Caloric Restriction

The **Calorie Restriction Diet (CR)** is the only dietary intervention proven to extend the healthspan and lifespan of primates [146,147]. CR involves restricting the calorie intake while maintaining full nutritional value. The effect of a one-year weight-loss diet with calorie restriction and exercise on inflammatory biomarkers was examined in 439 overweight and obese postmenopausal women. Diet alone and diet combined with exercise in women who lost 5% or more of their body weight exhibited reduced serum levels of CRP, IL-6, and amyloid A, inflammatory biomarkers [148]. Twenty-nine obese premenopausal women, after 12 weeks of calorie restriction by 500–1000 kcal per day along with a 50 g fiber supplement, demonstrated a 27% reduction in plasma IL-6 and IL-18 levels, with no significant change in IL-10 levels [149]. Two years of 25% CR in non-obese individuals resulted in a 10.4% weight loss and decreased circulating inflammatory markers, including total white blood cell count, lymphocyte count, ICAM-1, and leptin. Serum CRP and TNF-α levels were approximately 40% and 50% lower, respectively, in the CR group. No impairment of key markers of cellular immunity has been demonstrated [150]. Studies on primates have shown that the benefits of CR on T-cell function were evident only in animals that initiated the diet in adulthood; initiating it too early or too late negated these benefits [151]. Studies on mice and rats have shown that CR can reverse most of the signs of immune aging, such as defects in hematopoietic stem cells and changes in T-cell populations, including limiting the decline in the naïve lymphocyte pool and the accumulation of memory cells. CR can also improve the ability of T cells to proliferate and produce IL-2, which is crucial for immune signaling, and correct the age-related increase in serum levels of TNF-α and IL-6 inflammatory markers [152,153,154,155].

Calorie restriction by approximately 14% for 2 years in healthy humans significantly increased thymic volume and mass and reduced intrathymic adipose tissue, thereby improving T-cell production. At the molecular level, CR reduced *PLA2G7* gene expression. In the same study, the authors demonstrated that 24-month-old Pla2g7-deficient mice had a larger thymus and higher thymocyte abundance, and that they were protected from age-related thymic involution. Furthermore, Pla2g7 deletion in mice protected against inflammation, reduced NLRP3 inflammasome activation, and improved metabolic health. The authors suggested that in humans, CR-induced PLA2G7 reduction may contribute to improved adipose tissue metabolism, reduced inflammation, and reduced thymic lipoatrophy [30].

Severe dietary restriction by approximately 50% in mice reduced the pool of memory T cells in secondary lymphoid organs and blood but increased their accumulation in bone marrow. Adipogenesis, erythropoiesis, and increased concentration of trophic factors for resident T lymphocytes have also been observed in the bone marrow. The authors interpreted the results as a mechanism that protects memory cells and helps the organism adapt to dietary challenges [156]. Palma et al. demonstrated in mice that CR, which does not lead to malnutrition, protects animals against Mycobacterium tuberculosis infection by reducing the generation of foam cells, which serve as a reservoir for the bacteria. CR promotes glycolysis, reduces fatty acid oxidation, and reduces mTOR pathway activity. Consequently, autophagy increases in immune cells infected with microbes [38]. Moreover, Nakayama et al. demonstrated that 48 h of nutritional deprivation in young mice reduced thymus size, an effect not observed in aged mice. Furthermore, it increased the number of IgG^+^ cells only in aged animals; disrupted the structure of the thymic cortex–medulla boundary; and promoted a Th2 profile characterized by IL-5, IL-6, and IL-10 in both age groups, whereas aging itself induced a Th1 profile associated with IFN-γ secretion. In young mice, nutritional deprivation increased the expression of prostanoid-synthesizing enzymes, whereas in aged mice, this mechanism was attenuated [157]. Gardner et al. showed that aged mice infected with the influenza virus had lower NK cell activity and delayed viral clearance while fed CR compared to mice fed ad libitum [158].

Although CR appears to delay aging at the cellular level and improve some immune parameters measured in vitro, data on its effect on the whole organism’s ability to fight acute infections are equivocal. It is also still unknown what degree of caloric restriction will provide the greatest benefit. Dietary interventions such as intermittent fasting and the fasting-mimicking diet (FMD) are emerging as potentially effective in reducing inflammation and improving immune response. However, they can have both positive and negative effects on immunity, depending on the duration of CR and the composition of the diet. A cross-sectional study on 50 healthy volunteers (21 men and 29 women) observing Ramadan intermittent fasting revealed significant reductions in IL-1β, IL-6, and TNF-α compared with the pre-fasting baseline, accompanied by decreases in total leukocytes, monocytes, and granulocytes [159]. This study provided direct human evidence that prolonged intermittent fasting can attenuate circulating pro-inflammatory cytokine levels. Brandhorst et al. considered FMD involving bi-monthly cycles of 4 days of very low-calorie intake in mice and noted a positive effect on the regeneration of immune cells dependent on haematopoietic stem cells. Massive apoptosis of senescent immune cells was observed. A reduction in IGF-1 levels activated dormant hematopoietic stem cells in the bone marrow to rapidly divide. During the refeeding phase, the activated cells differentiated, significantly increasing the number of young lymphocytes and renewing the immune system. A reduction in circulating insulin-like growth factor 1 (IGF-1) levels partially reversed signs of age-related immunosenescence. The FMD was also associated with a reduced incidence of cancer, as well as a delay in tumor progression. Finally, repeated FMD cycles have been shown to extend median lifespan and delay the onset of age-related pathologies, thereby supporting its potential role in promoting healthy aging and longevity. Notably, in the same study, a human pilot trial involving 19 individuals, who completed three monthly cycles of a 5-day low-calorie FMD, showed significant reductions in multiple aging-related risk factors, including body weight, visceral fat, fasting glucose, and IGF-1. The study also noted improvements in blood pressure and markers of inflammation, such as CRP [160]. Calorie restriction and intermittent fasting studies demonstrating effects on immune function and inflammatory markers are presented in Table 3.

Nevertheless, confirming immune-specific effects in humans requires larger randomized controlled trials. Note that the immunological effects of caloric restriction may vary depending on the type of CR, the duration of the intervention, and the dietary composition. Moreover, the benefits of CR in primates have been primarily observed when this intervention was initiated in adulthood [151], while evidence from human randomized controlled trials remains limited.

## 4. Physical Activity

Physical activity is among the most promising non-pharmacological interventions to counteract immunosenescence [161]. Regular exercise has been shown to modify both innate and adaptive immune responses, potentially delaying or even reversing several hallmarks of immune aging [162]. The relationship between exercise and immune function follows a complex dose–response pattern, where moderate to vigorous physical activity confers benefits. In contrast, excessive exercise without adequate recovery may temporarily suppress immune function [163].

Physical exercise influences immune aging through multiple complementary mechanisms. Exercise induces acute mobilization of immune cells, particularly NK cells and cytotoxic T lymphocytes, from peripheral tissues into circulation [163]. This exercise-induced lymphocytosis, followed by post-exercise lymphocytopenia, reflects a redistribution of immune cells to peripheral tissues rather than immune suppression and has been proposed to enhance immune surveillance, potentially contributing to the attenuation of immunosenescence [164]. Regular physical activity throughout life is associated with a higher proportion of peripheral naïve T lymphocytes and a more favorable naïve/memory T-cell profile in older adults, suggesting that an active lifestyle may attenuate some age-related changes in T-cell composition linked to immunosenescence [165]. Exercise has been shown to modulate epigenetic mechanisms such as DNA methylation and histone modifications, leading to changes in gene expression profiles that include pathways involved in inflammation and immune regulation [23]. Regular physical activity is also associated with a reduced accumulation of senescent and exhausted T-cell subsets, including cells expressing inhibitory receptors such as programmed cell death protein 1 (PD-1) and may promote a more functionally competent T-cell profile in physically active individuals [166]. During exercise, acute bouts of muscle contraction induce transient increases in circulating cytokines such as IL-6, IL-8, IL-10, and the IL-1 receptor antagonist (IL-1ra), which act as signaling molecules and contribute to the systemic modulation of immune functions, including cytokine profiles, leukocyte trafficking, and inflammatory regulation [167].

The dose–response relationship between exercise and immune function follows a J-shaped curve, where moderate exercise enhances immunity, while excessive training may temporarily suppress immune function [168]. In elderly individuals, regular moderate-intensity exercise interventions performed consistently over months (e.g., combined aerobic and resistance training for 6 months) have been shown to improve immunological parameters, including increases in T-cell counts (e.g., CD3^+^, CD4^+^, CD8^+^) and favorable modulation of inflammatory cytokines, indicating potential enhancement of immune function with sustained training [169]. In older adults, moderate-intensity continuous training may be associated with more favorable alterations in selected T-cell subsets than high-intensity interval training. Given age-related changes in immune function, regular moderate exercise appears particularly relevant for older individuals, as it may help counteract aspects of immunosenescence [162].

**Aerobic exercise** has been shown to have significant immunomodulatory effects on elderly populations. Regular, moderate-intensity, continuous, aerobic training (MICT), and other forms of sustained cardiovascular activity increase the number and proportion of naïve T-cells while reducing the number of senescent, terminally differentiated, effector memory T-cells that re-express CD45RA (TEMRA) [170], which is a hallmark of immune aging [171]. A 12-week aerobic MICT intervention in older women at risk of breast cancer demonstrated that it increased the number of CD8^+^ effector memory T cells and elevated total lymphocyte counts. It also altered the CD4^+^/CD8^+^ ratio [172], implying a shift towards a healthier, more balanced immune profile [173]. These cellular changes suggest that aerobic exercise can partially restore the age-depleted naïve T-cell repertoire, which is critical for responding to novel antigens [169]. Furthermore, aerobic training significantly reduces systemic markers of inflammation. For example, six months of aerobic exercise in elderly subjects (61–66 years) significantly increased CD3^+^, CD4^+^, and CD8^+^ T-cell counts, decreased the CD4^+^/CD8^+^ ratio, and reduced circulating pro-inflammatory cytokines, including IL-6 and TNF-α [169]. In a supervised 12-week aerobic training intervention among middle-aged and older women, high-intensity interval training (HIIT) was associated with reductions in total CD4^+^ T cells and naïve CD4^+^ T-cell subsets. In contrast, moderate-intensity continuous training led to increases in total lymphocytes and effector memory CD8^+^ T cells compared with HIIT, indicating divergent effects of training intensity on peripheral T-cell profiles [170]. Twelve-week aerobic training programs have been shown to decrease circulating levels of IL-6, IL-8, and IL-10, suggesting a comprehensive immunomodulatory response [174]. Therefore, aerobic exercise dampens the chronic low-grade inflammation characteristic of aging [175]. In addition, even brief exercise triggers a rapid increase in the number of leukocytes in the bloodstream, followed by their movement into peripheral tissues, where they support immune surveillance and tissue repair. Over time, regular training produces lasting adaptations, resulting in a more robust and efficient immune system with improved cell trafficking and responsiveness. In older adults, habitual physical activity also helps preserve the migratory capacity of neutrophils, which typically declines with age [173]. Additionally, aerobic exercise enhances vaccine responses in elderly individuals, leading to a stronger antibody response to influenza vaccination than in sedentary individuals [175].

**Resistance training** offers distinct yet complementary benefits for immune function in aging, complementing aerobic exercise. Strength endurance training (SET), such as leg press, chest press, and seated rowing movements with high repetition numbers at moderate intensity (40% one-repetition maximum, an equivalent to approximately 40% of the maximum weight a person can lift in a single repetition), has proven particularly effective in older women in reducing the number of T cells that display markers of cellular aging and have lost proliferative capacity, with the magnitude of reduction positively correlating with the number of training sessions performed [176]. Interestingly, the effects of resistance training appear to depend on training modality. Intensive strength training (IST), conducted at 80% one-repetition maximum for 3–10 repetitions per set and typically performed 2–3 times per week, and SET have been observed to elicit immunological responses [177]. This suggests that a balanced program incorporating both intensive and endurance-type resistance training may optimize immune adaptations in older adults. Resistance training also demonstrates robust anti-inflammatory effects. Twelve weeks of high-intensity resistance exercise performed by older women 2–3 times per week at 60–85% of one-repetition maximum reduced CRP by 33%, leptin by 18%, and TNF-α by 29%, simultaneously improving muscle strength by 44% [178]. These changes occurred independently of changes in body composition, suggesting direct immunomodulatory mechanisms beyond fat mass reduction.

**Combined aerobic and resistance training** may offer superior benefits compared to single-modality exercise programs. A 12-week combined strength and endurance training program in subjects aged 50–70 attenuated the accumulation of CD8^+^ effector memory CD45RA^+^ (EMRA) T-cells, which remained unchanged in control groups [179]. The intervention also redirected the kynurenine pathway toward the production of kynurenic acid (KA), which is a neuroprotective and anti-inflammatory metabolite of tryptophan that can reduce chronic inflammation and insulin resistance by blocking NLRP3 inflammasome activation and influencing GPR35 receptors [31,180] Six-week low-dose combined resistance and endurance training significantly improved the CD4^+^/CD8^+^ T-cell ratio and reduced systemic levels of pro-inflammatory IL-6 and IL-8, anti-inflammatory IL-10, and vascular endothelial growth factor (VEGF) in elderly participants (mean age: 70.3 years) [174]. Notably, these benefits occurred with relatively short-term, low-threshold training, making combined exercise programs particularly accessible for older individuals with limited physical capacity.

Water-based combined training appears particularly effective at reducing markers of inflammaging. Twenty-eight weeks of combined aquatic exercise (aerobic and resistance components) performed three times per week in elderly participants significantly decreased TNFα levels across all training groups and increased IL-10 in one group, resulting in a favorable shift in the TNFα/IL-10 ratio [181].

Based on current evidence, regular physical activity should be regarded as a cornerstone intervention for maintaining immune competence in aging populations, as it counteracts key hallmarks of immunosenescence, enhances immune cell function, and promotes a more youthful immune phenotype in older adults [182]. Specifically, in older adults, multimodal exercise programs that combine moderate-intensity aerobic training with resistance exercise are associated with improvements in inflammatory markers and immune cell function, suggesting that combined training may provide broader immunological benefits than single-modality interventions [163]. Importantly, exercise-induced myokine signaling, particularly via IL-6 released from contracting skeletal muscle, contributes to systemic anti-inflammatory effects and improved immune regulation in individuals with chronic diseases [183].

However, the interpretation of current findings is constrained by the significant heterogeneity of exercise protocols, which prevents the comparison and establishment of a universal dose–response relationship between physical activity and immune function. Consequently, future research should focus on identifying optimal exercise prescriptions for specific immune outcomes, understanding the long-term sustainability of exercise-induced immune improvements, and elucidating the molecular mechanisms that translate physical activity into immune rejuvenation. Additionally, comparative studies examining the relative benefits of conventional exercise versus mind–body practices such as yoga across different immune parameters would help refine exercise recommendations for aging populations. A summary of selected clinical trials on physical activity and immune aging outcomes is provided in Table 4.

## 5. Stress Reduction

### 5.1. Neuroendocrine Immune Pathways of Chronic Stress

Chronic stress is one of the most significant modulators of immune function across the lifespan, with profound implications for the aging of the immune system. The relationship between psychological stress and immunosenescence has become increasingly recognized as a critical factor in understanding age-related immune decline and susceptibility to disease in humans, as high levels of perceived stress are associated with impaired immune function, increased oxidative and inflammatory stress, and immunosenescence-like changes in immune parameters [184]. As the global population ages, identifying effective stress reduction strategies to mitigate stress-related immune dysregulation has become increasingly important for promoting healthy aging and reducing the burden of age-related diseases. Chronic stress-induced alterations in immune function operate through complex neuroendocrine immune pathways, particularly involving dysregulation of the hypothalamic–pituitary–adrenal (HPA) axis and sustained cortisol signaling, which can promote immune imbalance, altered cytokine production, and pro-inflammatory responses [185]. Chronic physiological stress has been associated with changes in peripheral T lymphocyte subsets that resemble aspects of immunosenescence, including reduced frequencies of naïve T cells and altered distributions of regulatory T cells, which are characteristic immune aging phenotypes [186]. Understanding how stress reduction interventions modulate neuroendocrine and immune pathways offers promising opportunities for mitigating stress-related immune dysregulation. The HPA axis responds to psychological and physical stressors by releasing corticotropin-releasing hormone (CRH) from the hypothalamus, which stimulates adrenocorticotropic hormone (ACTH) secretion from the pituitary gland, ultimately leading to cortisol release from the adrenal cortex [187]. Simultaneously, psychological stress activates the sympathetic nervous system, leading to the release of epinephrine and norepinephrine, which modulate immune cell function through β-adrenergic receptors and promote pro-inflammatory gene expression in circulating leukocytes [188].

Acute stress responses are generally adaptive and can enhance immune surveillance by redistributing leukocytes to peripheral tissues, thereby preparing the organism for potential injury or infection [189]. Chronic psychological stress has been associated with persistent elevations in pro-inflammatory cytokines, particularly IL-6, suggesting a shift toward a sustained inflammatory phenotype [190]. In parallel, chronic stress has been linked in experimental and clinical studies to reduced NK cell cytotoxicity and impaired antibody responses to vaccination, indicating alterations in both innate and adaptive immune parameters [191]. Rather than inducing immunosuppression, chronic stress appears to cause immune dysregulation characterized by reduced immune surveillance and low-grade, chronic inflammation [15]. Although direct evidence of stress-induced senescent cell accumulation in human organs remains limited, observational studies indicate that greater exposure to chronic psychosocial stress is associated with increased expression of senescence-related markers, including p16^INK4a^, in peripheral blood mononuclear cells [192]. Given that senescent cells acquire a SASP characterized by the release of cytokines such as IL-6 and TNF-α, stress-related activation of senescence-associated pathways may contribute to the amplification of systemic inflammation and immune dysregulation observed with aging. Evidence summarized by Glaser and Kiecolt-Glaser indicates that stress may impair antibody responses to vaccination and increase antibody titers to latent herpesviruses (e.g., Epstein–Barr virus). Psychological stress has also been associated with delayed wound healing, underscoring its broad impact on both systemic and local immune processes [193].

At the cellular level, chronic psychosocial stress has been linked to accelerated telomere attrition in leukocytes over time, reflecting a biological aging phenotype in immune cells. In a well-characterized cohort followed over 10 years, increases in chronic stress were associated with greater leukocyte telomere shortening, independent of age and other confounders [194]. Furthermore, chronic psychosocial stress has been shown to alter immune regulation through sustained neuroendocrine activation. In a human cohort exposed to long-term social stress, immune cells exhibited reduced sensitivity to glucocorticoid signaling, a phenomenon described as glucocorticoid receptor resistance [195]. This impaired responsiveness limited the anti-inflammatory effects of glucocorticoids and was associated with increased expression of pro-inflammatory genes [195]. Chronic stress is associated with mitochondrial dysfunction and increased ROS production. Elevated ROS levels promote oxidative damage to mitochondrial DNA and mitochondrial membranes, disrupting oxidative phosphorylation and reducing ATP production, which ultimately impairs metabolic processes in lymphocytes and other immune cells [196]. Together, these neuroendocrine changes: sustained cortisol release, glucocorticoid receptor resistance, and sympathetic nervous system activation converge on NF-κB upregulation, promoting SASP production and contributing to the systemic inflammaging characteristic of immune aging.

### 5.2. Stress Reduction Techniques

**Mindfulness-based stress reduction (MBSR)** is one of the most widely studied psychological interventions for stress management. The standard MBSR program typically includes mindfulness meditation, body awareness exercises, and gentle yoga practiced over eight weeks. Evidence suggests that MBSR can influence immune function. For example, participants undergoing MBSR training have been shown to exhibit increased antibody titers following influenza vaccination, indicating enhanced adaptive immune responses [197]. Furthermore, mindfulness-based interventions, including MBSR, have been associated with reductions in inflammatory biomarkers such as CRP and pro-inflammatory cytokines, particularly in individuals experiencing elevated psychological stress [198]. The mechanisms underlying MBSR’s effects on immune function likely involve multiple neuroendocrine pathways. Mindfulness practice has been shown to modulate HPA axis activity and alter cortisol responses to stress, including enhanced cortisol habituation to repeated stressors. Such changes in glucocorticoid signaling may contribute to improved immune regulation [199]. Additionally, mindfulness-based and other mind–body interventions may influence inflammatory processes at the genomic level. Evidence from randomized trials indicates reduced NF-κB activity and decreased expression of inflammation-related genes following such interventions [28]. Thus, the immune effects of mindfulness practice operate not only through the neuroendocrine pathway, in which HPA axis dysregulation and glucocorticoid receptor resistance lead to NF-κB upregulation, but also through an epigenetic pathway, in which meditation reduces expression of HDAC2, HDAC3, and HDAC9, thereby affecting gene expression, e.g., by attenuating the transcription of pro-inflammatory genes, including RIPK2 and COX-2 [24].

Beyond MBSR, mindfulness **meditation interventions** have been associated with several immunological changes. Randomized controlled trials reviewed by Black and Slavich (2016) report alterations in inflammatory biomarkers such as CRP and IL-6, the modulation of pro-inflammatory transcription pathways, including the NF-κB pathway, and changes in cellular immune markers such as CD4^+^ T-cell counts and telomerase activity [200]. Molecular studies suggest that meditation may influence immune-related gene expression and epigenetic regulatory pathways. In peripheral blood mononuclear cells from experienced meditators, a day of intensive mindfulness practice was associated with reduced expression of several histone deacetylase genes, including *HDAC2*, *HDAC3*, and *HDAC9*, as well as decreased expression of pro-inflammatory genes such as *RIPK2* and *PTGS2* (*COX-2*). These genes are involved in inflammatory signaling pathways and in chromatin remodeling processes that regulate immune gene transcription. In addition, the downregulation of *HDAC2* and *RIPK2* expression has been associated with faster cortisol recovery following a psychosocial stress test, suggesting a link between epigenetic regulation, stress physiology, and inflammatory gene expression. These findings indicate that mindfulness meditation may modulate immune function through epigenetic mechanisms affecting inflammatory transcriptional pathways [24].

**Cognitive–behavioral stress management (CBSM)** integrates cognitive restructuring, coping skills training, and relaxation techniques aimed at reducing psychological stress. In a randomized controlled trial among women undergoing treatment for breast cancer, CBSM was associated with significant alterations in leukocyte gene expression profiles. Specifically, the intervention resulted in the downregulation of pro-inflammatory gene transcripts and the upregulation of type I interferon response genes. Promoter-based bioinformatic analyses further indicated reduced activity of NF-κB/Rel and GATA transcription factors alongside increased activity of interferon response factors and the glucocorticoid receptor, suggesting a shift toward a lower inflammatory transcriptional profile in circulating immune cells [29].

**Yoga** is an ancient mind–body practice combining physical postures (asanas), breathing techniques (pranayama), and meditation that has emerged as an effective intervention for modulating immune function in older adults [201]. Yoga emphasizes breath regulation, mindfulness during practice, and the maintenance of postures, which distinguish it from standard physical exercise [202]. Gallegos et al. demonstrated that components of an 8-week MBSR program, particularly yoga and sitting meditation, were significantly associated with higher post-treatment IGF-1 levels [203]. An eight-week Hatha yoga intervention conducted three times per week by patients with allergic rhinitis resulted in significant improvements in peak nasal inspiratory flow (PNIF), reductions in nasal blood flow, and increased nasal secretion of IL-2. These findings suggest a potential modulatory effect of yoga on nasal physiological function and local immune response in individuals with allergic rhinitis [204]. A comprehensive meta-analysis of randomized controlled trials evaluated mind–body interventions, including yoga, Tai Chi, Qi Gong, and meditation, and reported a moderate reduction in CRP following interventions lasting 7 to 16 weeks. Small but significant increases were also observed in CD4+ T-cell and NK cell counts. In contrast, changes in other inflammatory markers, such as IL-6, TNFα, IL-1β, and IFNγ, were generally small and not significant [205]. In a randomized controlled study on elderly nursing home residents, relaxation techniques were associated with specific changes in immune cell markers. Immediately after the intervention, the treatment group exhibited a significant increase in CD71 (transferrin receptor) and CD97 expression, both of which indicate higher lymphocyte activation and proliferative activity. At the same time, there were significant decreases in the proportion of CD19+ B lymphocytes and in CD134 (OX40) expression on activated T cells, compared with pre-treatment levels. These changes were significant at post-treatment and, for some markers (CD19, CD71, CD97), persisted at the three-month follow-up [206]. Other studies have suggested that yoga may be associated with reductions in circulating stress and inflammatory biomarkers. Several studies have reported decreases in cortisol and pro-inflammatory markers, including CRP, IL-6, TNFα, and IFNγ. Research suggests that yoga-based lifestyle interventions (YBLIs) may influence molecular markers associated with inflammation, oxidative stress, and aging in obese adults. In a 12-week randomized controlled trial, participants undergoing YBLI showed early increases in telomerase reverse transcriptase (TERT) expression in peripheral blood mononuclear cells at 2 weeks; however, this difference was not sustained at the end of the 12-week period. Also, while TNF-α levels showed temporal variability, no consistent long-term reduction was observed after the intervention. Additionally, no significant between or within-group changes in IL-6 or NF-κB were observed over 12 weeks [207].

Growing evidence suggests that yoga may modulate immune function by altering inflammatory pathways. This pattern is reflected in the systematic review by Yeun and Kim [208]), which analyzed 11 randomized controlled trials examining immune outcomes. The most frequently assessed markers were IL-6, TNF-α, and CRP. Several studies reported significant reductions in IL-6 following yoga interventions, while decreases in TNF-α and CRP were observed in some trials, though not all. A smaller number of studies have also evaluated other cytokines; in these cases, pro-inflammatory markers such as IL-1β and IL-17A tended to decline, whereas the anti-inflammatory cytokine TGF-β increased in certain clinical populations. Due to heterogeneity in study design, participant characteristics, and intervention protocols, the findings were synthesized narratively, and the overall effects were found to be inconsistent across studies [208]. However, these effects were not observed in all studies and may vary depending on the type of yoga, the study population, and the methodology used [201].

**Social support and social integration** are associated with lower levels of inflammation. A meta-analysis of 41 studies found that stronger social relationships are associated with lower levels of inflammatory biomarkers, including IL-6 and CRP. These markers reflect systemic inflammatory activity, suggesting that inflammation may be one biological mechanism linking social relationships to health outcomes [209]. Social isolation and relationships have been linked to the dysregulation of immune-related processes. Evidence from psychoneuroimmunology research indicates that individuals with poorer social relationships or higher relational stress may exhibit weaker antibody responses to vaccination, slower wound healing, and higher levels of inflammatory markers [210]. A synthesis of 23 meta-analyses encompassing 1187 studies and over 1.4 billion participants demonstrated that supportive social relationships exert their protective effects partly through downregulation of HPA axis activity and attenuation of cortisol responses to stress, providing a direct neuroendocrine link between social integration and immune health [211]. The biological mechanism underlying these associations involves the sympathetic nervous system. Psychosocial stress triggers the release of norepinephrine, which binds β-adrenergic receptors on leukocytes and activates NF-κB-driven pro-inflammatory gene expression [212]. Structured social engagement may attenuate this pathway by reducing sympathetic tone and perceived threat. Social prescribing, the formal referral of patients to community-based social activities, represents a structured clinical approach to harnessing these benefits and has been implemented in national healthcare systems in the United Kingdom, the Netherlands, and Japan [213].

A major challenge for stress reduction interventions is achieving sustained adherence. Many studies demonstrate benefits during active intervention phases, but long-term maintenance of practice and sustained effects are less well documented. The demands of regular meditation, exercise, or other practices may be difficult to maintain, particularly for older adults facing health challenges or lacking social support. Future research should explore precision medicine approaches to stress management for immune health, identifying biomarkers that predict response to specific interventions and tailoring recommendations based on individual profiles. Genetic, epigenetic, and immunological assessments may help match individuals to the most appropriate stress reduction strategies. Large-scale, longitudinal studies are needed to definitively establish whether stress reduction interventions can slow immune aging trajectories and reduce age-related disease incidence. These studies should include comprehensive immune assessments, functional outcomes, and biomarkers of biological aging.

## 6. Quality Sleep

Sleep and stress are closely interconnected physiological processes, and sleep disturbance is associated with increased activation of inflammatory pathways. Experimental and epidemiological studies indicate that insufficient sleep promotes inflammatory signaling, including activation of NF-κB, and is associated with elevated levels of IL-6, TNF, and CRP. Evidence also shows that shorter sleep duration around the time of vaccination can reduce antibody responses to vaccines such as influenza and hepatitis A, and insufficient sleep has been linked to greater susceptibility to viral infections, including rhinovirus [214]. Behavioral interventions that improve sleep quality can influence inflammatory processes. Randomized clinical trials in older adults with insomnia have shown that cognitive–behavioral therapy for insomnia (CBT-I) is associated with reductions in systemic inflammation, including decreases in CRP and in TLR-4-stimulated production of pro-inflammatory cytokines by monocytes. These changes are accompanied by reduced expression of pro-inflammatory genes and decreased activity of NF-κB and AP-1, indicating that improving sleep may attenuate inflammatory signaling pathways relevant to health in older adults [215].

## 7. Cold Exposure

Cold exposure (CE), particularly whole-body cold-water immersion, can acutely modulate immune parameters. Experimental studies have shown that immersion in cold water (13–14 °C) induces a physiological stress response accompanied by marked changes in circulating white blood cell counts, with peaks occurring within 1–2 h after exposure. In addition, increases in pro-inflammatory cytokines such as IL-6 and IL-1β have been reported to occur with a delayed time course following cold-water immersion [216]. Prolonged cold exposure induces anti-inflammatory changes in adipose tissue. In rats exposed to continuous cold (4 °C for up to 7 days), an anti-inflammatory response was observed in mesenteric white adipose tissue, characterized by macrophage infiltration, increased catecholamine production and thermogenin expression, and the inhibition of pro-inflammatory cytokine expression [217]. Acute cold exposure can transiently influence circulating immune cell populations. Experimental studies comparing cold-water immersion and partial-body cryotherapy have shown increases in circulating CD8^+^ T and NK cells following exposure. These changes primarily involve cytotoxic CD16^+^ NK cell subsets and are interpreted as a transient mobilization of lymphocytes into the circulation rather than increased cell proliferation [218]. In a large randomized controlled trial, individuals who ended their daily warm shower with 30–90 s of cold water for 30 days experienced a 29% reduction in work absence due to illness compared with controls. However, the total number of illness days did not differ significantly between groups [219]. Observational studies suggest that recreational cold-water immersion combined with specific breathing techniques may be associated with improved mental health indices and shorter upper respiratory tract infection duration compared with non-practitioners or individuals practising cold exposure alone [220]. In a randomized controlled trial, daily cold showers for 90 days were associated with significant increases in circulating IgG, IgA, and IgM and elevated levels of IL-2 and IL-4, indicating enhanced humoral and cell-mediated immune responses [221]. A three-week repeated cold-water immersion intervention (7 °C for 12 min, four times per week) in healthy men resulted in a significant decrease in circulating neutrophil number and proportion, while no significant changes were observed in other leukocyte subpopulations [222]. Other studies have also shown that acute cold exposure can alter circulating leukocyte counts and subsets, including increases in total leukocytes, granulocytes, and NK cells. These responses are thought to reflect a transient mobilization of immune cells associated with sympathetic activation during cold stress [223]. A comprehensive review found that cold-water immersion incurs time-dependent effects on inflammation, stress, immunity, sleep quality, and quality of life, with significant increases in inflammation immediately and 1 h post-exposure, followed by stress reduction at 12 h post-exposure [224]. Longer-term benefits included improved sleep quality and reduced sickness absence, though the evidence base remains limited by small sample sizes and a lack of population diversity.

Despite promising findings, several important limitations and considerations must be acknowledged. First, individual responses to cold exposure vary significantly, depending on factors such as age, body composition, baseline health status, and prior cold adaptation. Second, the optimal parameters for CE (temperature, duration, frequency) that maximize immune benefits while minimizing risks remain incompletely defined. Third, most studies have examined relatively short-term interventions, and the long-term effects of regular CE on immunosenescence remain to be investigated. Additionally, CE carries potential risks, including hypothermia, cardiovascular stress, arrhythmias, and cold shock response, which may be problematic in individuals with pre-existing cardiovascular or respiratory conditions. Advanced age itself is associated with impaired thermoregulatory responses and increased vulnerability to cold-related injury [225]. Therefore, any CE intervention should be implemented cautiously and individualized based on each person’s health status and tolerance. Future research should focus on several key areas to better understand the relationship between cold exposure and immune aging. Longitudinal studies should be conducted to examine the effects of regular cold exposure on immunological markers and infection rates in aging populations. Additionally, mechanistic studies elucidating the molecular pathways by which cold exposure influences immune cell function, inflammation, and cellular aging would provide valuable insights. Standardized protocols for CE are also required. Such protocols should consider individual factors, including age, health status, cold adaptation level, and comorbidities.

## 8. Vaccination

Vaccination is one of the most effective tools for infectious disease prevention in older adults, and a growing body of research indicates that its effects may extend beyond direct protection against specific pathogens. One of the best-studied examples of the impact of vaccination on aging is the varicella-zoster virus (VZV) vaccine. This virus remains latent in the body and can reactivate in old age, causing shingles and exacerbating inflammatory processes. Kim and Crimmins studied a representative US sample of 3884 adults aged 70^+^ and found that vaccination against shingles was significantly associated with lower levels of inflammatory markers, slower epigenetic and transcriptomic aging in venous blood, and a lower overall biological aging index. These effects were most pronounced within three years of vaccination but persisted beyond that period [226]. One proposed mechanism for this phenomenon is the reduction in latent VZV reactivation. Viral reactivation leads to increased production of inflammatory cytokines such as IL-6 and TNF-α, which may accelerate inflammatory processes and biological aging. Besides direct protection against infection, vaccines may also affect the overall immune system (immune fitness) by activating both innate and adaptive immune responses. These mechanisms include, among others, inhibiting immune responses, improving antigen presentation, activating T lymphocytes, and increasing antibody production [227,228,229]. A growing body of research indicates that vaccinations can also induce so-called trained immunity, i.e., long-term reprogramming of innate immune cells. This involves epigenetic and metabolic changes in cells such as monocytes and macrophages, which lead to a stronger immune response upon subsequent contact with pathogens [227,230,231]. This mechanism may partially compensate for the age-related decline in immune function and reduce susceptibility to infections in the elderly population. The contemporary approach to preventive healthcare for older adults involves integrating vaccinations with other lifestyle factors. The concept of immunofitness views vaccination as a tool to support immune balance and reduce the risk of infectious complications [232,233]. Epidemiological studies also indicate that vaccination against diseases such as influenza and pneumococcal disease may reduce the risk of hospitalization, cardiovascular complications, and mortality in the elderly population.

## 9. Synergistic and Integrated Approaches

No single lifestyle intervention addresses all the mechanisms driving immune aging simultaneously, which is why combined, multicomponent strategies are more likely to produce meaningful clinical effects. Despite the clear rationale for this approach, trials testing combinations of interventions with immune-specific outcomes in older adults remain rare. This is partly a practical problem, as designing a trial that simultaneously controls diet, exercise, stress reduction, and sleep is considerably more complex than testing one intervention at a time, and isolating the contribution of individual components is difficult. In a pilot randomized control trial, Fitzgerald et al. enrolled 43 healthy men aged 50–72 years in an eight-week program combining dietary guidance, sleep support, supervised exercise, relaxation practice, probiotics, and phytonutrient supplementation. Compared with controls, participants showed a mean reduction of 3.23 years in epigenetic age, measured using the Horvath DNA methylation clock (*p* = 0.018) [234]. The primary outcome was biological age rather than immune parameters. Nonetheless, the finding shows that a well-designed multicomponent program can produce measurable biological effects within weeks, even in relatively healthy middle-aged and older adults. Based on the evidence reviewed, the most promising integrated strategy for attenuating immune aging combines an anti-inflammatory dietary pattern (Mediterranean or MIND diet), combined moderate-intensity aerobic and resistance exercise at least three to five days per week, evidence-based stress management such as MBSR or CBSM, seven to eight hours of sleep per night, age-appropriate vaccination, and targeted vitamin D, vitamin C, zinc, and omega-3 fatty acid supplementation where dietary intake is insufficient. This multicomponent approach engages all molecular pathways discussed in this review and represents the current framework for improving immune competence in older adults. Conducting large-scale trials of this kind remains challenging; however, as recruiting sufficient numbers of older adults, maintaining adherence to multiple simultaneous interventions over extended periods, and disentangling the specific contribution of each component all require considerable resources and careful trial design.

## 10. Summary

Immune system aging results from the progressive involution of lymphoid organs and functional alterations in immune cells, leading to immune dysfunction. Hallmarks of this process are chronic, low-grade systemic inflammation, inflammaging, driven in part by the accumulation of senescent cells and their pro-inflammatory secretory phenotype (SASP); impaired immune surveillance; increased susceptibility to infections; reduced vaccine effectiveness; increased risk of autoimmunity; and age-related diseases such as cardiovascular disease, type 2 diabetes, neurodegeneration, and cancer. Growing evidence indicates that lifestyle-based interventions can meaningfully mitigate these processes. Nutritional strategies, including anti-inflammatory dietary patterns such as the Mediterranean, DASH, and MIND diets, as well as caloric restriction and fasting-related approaches, can reduce systemic inflammatory markers, support immune cell function, and slow biological aging. Key dietary components with documented immunomodulatory properties include ω-3 fatty acids; polyphenols and flavonoids; vitamins C, D, and E; and zinc. Regular physical activity, particularly combined aerobic and resistance training at moderate intensity, has been shown to attenuate key hallmarks of immunosenescence, including the accumulation of senescent T-cell subsets, reduction in naïve T-cell pools, and chronic low-grade inflammation. Psychological stress reduction through evidence-based interventions such as MBSR and CBSM can modulate neuroendocrine immune pathways, reduce inflammatory gene expression, and attenuate stress-related immune dysregulation. Vaccination represents a complementary tool that not only provides direct protection against pathogens but may also support broader immune fitness through trained immunity mechanisms and reduction in chronic antigenic stimulation associated with latent viral infections. Emerging approaches, including mind–body practices and controlled cold exposure, show preliminary promise in modulating inflammatory markers and immune cell populations, although the current evidence base remains limited by methodological heterogeneity and small study populations.

Altogether, integrated lifestyle strategies targeting multiple pathways of immune aging simultaneously appear more effective than single-component interventions. However, significant challenges remain, including the need to identify optimal intervention parameters, define population-specific recommendations, and establish long-term efficacy through rigorously designed longitudinal studies. Therefore, the take-home message is that the molecular hallmarks of immune aging are addressed by different interventions through partially overlapping mechanisms, making multimodal strategies inherently superior.

### 10.1. Limitations of the Current Evidence

Despite the large number of studies confirming the immunomodulatory potential of lifestyle interventions, important limitations must be considered before these findings are implemented in clinical practice. A common challenge across all the interventions analyzed in this review is the small number of study participants, the short duration of the interventions, and the lack of long-term follow-up. For dietary interventions, most of the evidence comes from animal models or cell culture studies. Moreover, human data are usually collected through self-completed questionnaires, and observational data linking dietary patterns to immune markers are susceptible to confounding factors such as physical activity levels, socioeconomic status, and overall health behaviors. While studies on supplementation are more controlled, they often analyze individual nutrients, which do not reflect the complexity of real-world dietary patterns and nutrient interactions.

Although moderate CR can reduce inflammatory markers and may protect thymic function [30,150], the extent of these benefits depends on the degree of restriction, the age at which it is initiated, and the individual’s health status. In fact, CR can simultaneously lower inflammation and weaken the acute response to infection, depending on how it is applied. In primate studies, immunological benefits were observed only when CR was initiated in adulthood, not in very young or aged animals [151]. In frail or older humans, even moderate restriction carries a real risk of sarcopenia and nutritional deficiency, and the benefits observed in healthy, non-obese adults may not translate to these groups. CR should therefore only be recommended under medical supervision, with careful monitoring of infection susceptibility. In turn, severe restriction, meaning an approximately 50% reduction in caloric intake in mice, has been shown to redistribute memory T cells to the bone marrow [156] and impair NK cell activity, delaying viral clearance in aged animals [158].

Similarly, differences in exercise protocols, including their form, intensity, frequency, and duration, make it difficult to reach firm conclusions regarding the effects of physical exercise on the immune system. Furthermore, many studies have failed to account for important contributing factors, such as medication use and baseline physical condition, which can significantly impact the immune response to training.

In addition, while promising, the evidence regarding yoga and other mind–body practices remains inconsistent across studies, probably due to variability in how these interventions are implemented. Stress reduction interventions, in turn, face challenges in measuring stress levels and psychological resilience, as these are inherently subjective and difficult to standardize. Studies differ in how they define and assess these factors, and findings regarding the immune system are rarely comparable across studies.

Similarly, evidence on interventions to improve sleep quality largely comes from populations with clinical insomnia. The question of whether improving sleep quality in older adults without overt sleep disorders yields similar benefits for the immune system remains unanswered.

Cold exposure currently has the weakest evidence base of all interventions reviewed. Most trials consider only the short term, are conducted in healthy young adults, and use widely varying protocols. Acute immune changes, such as transient mobilization of NK cells and cytotoxic T lymphocytes, have not been shown to translate into sustained improvements in immune competence or resistance to infection in older populations. Moreover, the risks of cold exposure in older adults, including cardiovascular stress and impaired thermoregulatory responses, are real and must not be minimized.

In turn, vaccination is one of the interventions with the best-documented efficacy. Even in this case, however, the mechanism linking vaccine-induced changes to long-term trends in immune system aging still needs to be confirmed with prospective data from longitudinal studies.

An important limitation of all the interventions included in this review is the limited number of clinical trials involving older adults with frailty, individuals with multiple comorbidities, and people from diverse ethnic and socioeconomic backgrounds. Most studies recruit relatively healthy, motivated volunteers, creating a discrepancy between the study populations and the groups that, in clinical practice, are most likely to benefit from lifestyle interventions supporting the immune system. Another limitation that has received little attention in the reviewed literature is the potential for interactions between lifestyle interventions and medications commonly prescribed to older adults. Many older patients take statins, metformin, or other antidiabetics, antihypertensives, or immunosuppressants, each with effects on inflammatory signaling, mitochondrial function, and immune cell activity. Whether these pharmacological backgrounds modify, enhance, or attenuate the immune effects of lifestyle interventions is largely unknown, as most trials either exclude medicated participants or fail to report medication use in sufficient detail. This is a clinically important gap, since those who would benefit from lifestyle interventions the most are older individuals, likely on multiple medications.

A related limitation concerns the selection of biomarkers. The reviewed literature does not provide a consensus on which immune or inflammatory markers best reflect the clinical changes associated with immunosenescence. Based on the evidence analyzed here, the most reliable biomarkers appear to be circulating IL-6 and TNF-α, C-reactive protein, the ratio of naϊve T cells to T-TEMRA cells in peripheral blood, and thymus volume assessed via MRI as an indicator of residual T-cell production capacity. The standardization of these biomarkers in future interventional studies would significantly improve comparability across studies and enable reliable meta-analyses.

Not all interventions discussed in this review are equally well supported by human clinical evidence, and this distinction matters when translating findings into practice. A summary of the evidence strength for each intervention category is provided in Table 5, which ranks interventions by study type, sample size, evidence grade, and consistency of findings across studies. In terms of the relative strength of human clinical evidence, interventions can be ranked as follows: (1) Mediterranean diet, supported by multiple large RCTs, including PREDIMED; (2) vitamin D supplementation with the VITAL trial, demonstrating 22% reduction in autoimmune disease incidence; (3) moderate aerobic and combined exercise, exhibiting consistent evidence through multiple RCTs in older adults; (4) caloric restriction through the CALERIE-2 trial; (5) MBSR via multiple validated RCTs; and (6) vaccination (VZV, influenza), demonstrating strong epidemiological and prospective cohort evidence. Mind–body practices and cold exposure currently have the weakest evidence base in human clinical trials. This hierarchy should inform clinical prioritization when recommending integrated lifestyle strategies to older patients.

Altogether, these limitations highlight the need for more rigorous, standardized clinical trials with longer follow-up periods, more diverse and clinically representative populations, and consistent outcome measures that allow meaningful comparisons across studies and intervention types.

### 10.2. Future Research Directions

Large-scale, long-term randomized controlled trials are needed to evaluate the effects of combined lifestyle interventions in diverse older adult populations, including those who are frail or have multiple chronic conditions and groups who are currently under-represented in the literature. Greater consistency in the immune and inflammatory biomarkers measured across studies would also enable direct comparisons and pooled analyses that are currently not possible. Understanding why some individuals respond well to a given lifestyle intervention while others show minimal benefit is also important. Addressing this will require studies that integrate clinical, genetic, and immunological data at the individual level. The long-term effects of combined exercise and dietary interventions on immune cell gene regulation, including changes in DNA methylation and inflammatory gene expression, also warrant more systematic investigation in human cohorts. Finally, head-to-head trials comparing single-component approaches with comprehensive multimodal programs, using clinically relevant outcomes such as infection rates, vaccination responses, and hospitalization risk, would substantially strengthen the evidence base for lifestyle medicine in older adults.

## Figures and Tables

**Table 1:** Effects of selected dietary components on immune system cell function and inflammation. *AIRE: autoimmune regulator; CCL2: C-C motif chemokine ligand 2; COX: cyclooxygenase; CRP: C-reactive protein; CTL: cytotoxic T lymphocyte; DC: dendritic cell; IFN-γ: interferon gamma; IL: interleukin; LOX: lipoxygenase; NET: neutrophil extracellular trap; NF-κB: nuclear factor kappa-light-chain-enhancer of activated B cells; NK: natural killer; PI3K/Akt/mTOR: phosphoinositide 3-kinase/protein kinase B/mechanistic target of rapamycin; ROS: reactive oxygen species; sICAM-1: soluble intercellular adhesion molecule-1; sP-selectin: soluble P-selectin; sVCAM-1: soluble vascular cell adhesion molecule-1; Th: T helper lymphocyte; TNF-α: tumor necrosis factor alpha; Treg: regulatory T cell.*

Dietary Component	Effect on Immune System and Inflammation
**VITAMINS**	
**Vitamin C**	Neutralizes ROS Supports phagocytosis, chemotaxis, and microbial elimination by phagocytic cells Supports proliferation and differentiation of B and T lymphocytes Supports apoptosis and macrophage removal of used neutrophils from infection sites Increases the number of thymocytes and splenocytes Increases the number of leukocytes, lymphocytes, granulocytes, and monocytes in peripheral blood Increases the number of naïve T lymphocytes in peripheral blood Increases the number of clusters of differentiation CD4^+^CD8^+^, or CD4^+^CD8^−^ or CD4^−^CD8^+^ T cells in the thymus Provides antiviral, antibacterial, antiparasitic, and antifungal effects
**Vitamin E**	Increases cutaneous immune response to antigen Increases lymphocyte activity due to enhanced IL-2 production Increases lymphocyte mitogenic response Reduces plasma lipid peroxide concentration and prostaglandin E2 synthesis by peripheral blood mononuclear cells
**Vitamins C + E**	Provides vitamin C and vitamin E separately Increases production of IL-1β and TNF-α Decreases production of prostaglandin E2 Induces a tolerogenic phenotype in dendritic cells
**Vitamin D**	Increases antioxidant levels by interacting with Klotho and Nrf2 Induces expression of genes encoding antimicrobial peptides: cathelicidin antimicrobial peptide/LL-37, defensins, hepcidin, and neutrophil peptides Reduces TNF-α Increases NK cell cytotoxicity Increases neutrophil recruitment Inhibits NF-κB Delays dendritic cell differentiation, inhibits antigen presentation, and limits DC-dependent T-cell activation and proliferation Reduces expression of the Th17 lymphocyte subset Increases expression of Treg Increases levels of IL-4, IL-5, IL-10, and CCL2 anti-inflammatory cytokines Inhibits B-cell proliferation and immunoglobulin production Stimulates autophagy Participates in the establishment of central tolerance by increasing AIRE expression
**MINERALS**	
**Zinc**	Enhances neutrophil chemotaxis Supports cytotoxic activity of NK cells, the reactivity of B and T cells, the phagocytic properties of macrophages, and the functioning of dendritic cells Enhances Treg activity Increases naïve T lymphocyte population Balances Th1/Th2 response Maintains balanced cytokine levels and B lymphocyte function
**DIETARY LIPIDS**	
**ω-3 fatty acids**	Reduce the production of eicosanoids from arachidonic acid Produce anti-inflammatory compounds (resolvins, protectins) Exhibit anti-inflammatory effects due to changes in the composition of membrane phospholipids and their influence on the formation of lipid rafts in the cell membrane Inhibit NF-κB activation Maintain mitochondrial function
**Extra virgin olive oil**	Possesses Antioxidant and anti-inflammatory properties Reduces plasma levels of sICAM-1, sVCAM-1, sP-selectin, IL-6, and CRP Optimizes the lipid profile of mitochondrial membranes Reduces the expression of CD49d and CD40 on T lymphocytes and of CD49d and CD40 on monocytes Reduces the atherosclerotic plaque stability parameters of IL-18 and the IL18/IL10 ratio
**POLYPHENOLS AND FLAVONOIDS**	
**Polyphenols and flavonoids**	Inhibit ROS Inhibit pro-inflammatory cytokine production Inhibit protein kinases involved in signaling during inflammation Induce autophagy in macrophages through the IL-6/STAT3/FOXO3 pathway Inhibit the COX1/2 and 5-LOX pathways Increase proliferation, viability, activation, and cytotoxicity of NK cells Inhibit dendritic cell maturation and differentiation Promote proliferation of Treg lymphocytes Increase activity of CTLs Inhibit Th lymphocyte activation or proliferation Inhibit M1 macrophage activation and promote conversion to the anti-inflammatory M2 phenotype Inhibit NF-κB Reduce the ability of neutrophils to form NETs, increasing their bactericidal activity Anticancer effects: Convert M2 macrophages to pro-inflammatory M1 phenotype Increase activation and proliferation of CD8^+^ T lymphocytes Increase production of pro-inflammatory cytokines (e.g., IFN-γ) Reduce the number of myeloid-derived suppressor cells in the tumor microenvironment, inhibition of their immunosuppressive functions, and differentiation toward mature, less suppressive dendritic cells Inhibit the PI3K/Akt/mTOR axis and induction of apoptosis in tumor cells

**Table 3:** Selected caloric restriction and intermittent fasting studies demonstrating effects on immune function and inflammatory markers. *CR: caloric restriction; CRP: C-reactive protein; CVD: cardiovascular diseases; FMD: fasting-mimicking diet; IF: intermittent fasting; IL: interleukin; IGF-1: insulin-like growth factor 1; MRI: magnetic resonance imaging; NK cells: natural killer cells; PLA2G7: platelet-activating factor acetyl hydrolase (PLA2G7)-encoding gene expression; Pla2g7-KO: Pla2g7-deficient mice; RCT: randomized controlled trial; TNF-α: tumor necrosis factor α.*

Intervention	Type of Study	Sample Size	Target Organ/ System	Inflammaging Markers	Effect of Intervention	Ref.
**CR** with or without exercise *1 year*	Human RCT	N = 439 Obese postmenopausal women	Systemic, blood	CRP, IL-6, white blood cell count, and amyloid A	Decreased CRP, IL-6, and amyloid A levels in plasma; decreased neutrophil count in blood, 5% weight loss	[148]
**CR** 500–1000 kcal/day + fiber 50 g/day, *12 weeks*	Human RCT	N = 29 Obese premenopausal women	Systemic	IL-6, IL-18, IL-10	27% reduction in plasma IL-6 and IL-18; no significant change in IL-10	[149]
**25% CR** (CALERIE) *2 years*	Human RCT	N = 218 Non-obese adults	Systemic, lymphoid	CRP, TNF-α, ICAM-1, white blood cell count	CRP ≈ 40% lower, TNF-α ≈ 50% lower; preserved cellular immunity; no immune impairment	[150]
**14% CR** *2 years*	Human RCT + animal (mice)	CALERIE-2 subset (N = 220 total randomly selected non-obese adults; MRI imaging of the thymus); Pla2g7-KO aged mice	Thymus, adipose tissue	thymic volume, PLA2G7, NLRP3, inflammatory cytokines	Increased thymic volume; reduced intra-thymic fat; reduced PLA2G7 and NLRP3 activation	[30]
**25–35% CR** *28 days*	Animal (mice)	N = 96	Blood, lung, lymphoid organs, adipose	mTOR, autophagy	Increased resistance to tuberculosis by inhibiting mTOR and enhancing autophagy; reduced inflammation; improved T-cell response; increased survival	[38]
**IF** (Ramadan model)	Human observational	N = 50 Healthy volunteers (21 men and 29 women)	Systemic	IL-1β, IL-6, TNF-α, total leukocytes, monocytes, granulocytes, lymphocytes	Significant reduction in pro-inflammatory cytokines during the fasting period	[159]
**FMD** *5days/month*	Animal (mice) + human pilot	N = 19 Humans Multiple mouse cohorts	Bone marrow, systemic	IL-6, CRP, IGF-1, lymphocyte regeneration	Reduced risk factors for cancer, diabetes, and CVD; increased lymphocyte regeneration in mice	[160]
**Long-term CR**	Animal (rhesus macaques aged >20 years) prospective cohort	N = 76 Primates	Systemic, T-cell compartment	T-cell function, survival, age-related disease	Improved health and survival; T-cell benefits only when CR is initiated in adulthood, not in old age	[147,151]

**Table 4:** Selected studies on physical activity interventions and immune aging outcomes in older adults. *1RM: one-repetition maximum; CM: central memory T cells; CMV: cytomegalovirus; CRP: C-reactive protein; DXA: dual-energy X-ray absorptiometry; ELISA: enzyme-linked immunosorbent assay; EM: effector memory T cells; EMRA/TEMRA: effector memory T cells re-expressing CD45RA (terminally differentiated); HIIT: high-intensity interval training; HRmax: maximum heart rate; IL: interleukin; IL-1ra: interleukin-1 receptor antagonist; IST: intensive strength training; KA: kynurenic acid; LPS: lipopolysaccharide; MICT: moderate-intensity continuous training; QA: quinolinic acid; RCT: randomized controlled trial; RPE: rating of perceived exertion; RT: resistance training; RTE: recent thymic emigrants; SET: strength endurance training; TNF-α: tumor necrosis factor alpha; VEGF: vascular endothelial growth factor.*

Type of Activity	Study Type	Sample Size	Evaluation Method	Inflammaging Markers	Effect of Intervention	Ref.
**AEROBIC EXERCISE**						
**Aerobic** vs. **resistance** (60–80% HRmax or 60–80% 1RM) 40 min, 3×/week *6 months*	Human RCT	N = 80 (N = 40 aerobic; N = 40 resistance) elderly 61–66 years	Flow cytometry, ELISA	CD3+, CD4+, CD8+ T cells, CD4/CD8 ratio, IL-6, TNF-α, IL-10	Aerobic training increased CD3+, CD4+, and CD8+ T-cell counts and IL-10 and reduced IL-6 and TNF-α. CD4/CD8 ratio decreased in both groups. All effects were significantly greater in the aerobic than in the resistance group.	[169]
**MICT** vs. **HIIT** 3 sessions/week *12 weeks*	Human RCT	N = 24 post-menopausal women at high breast cancer risk	Flow cytometry VO2max testing	CD4+, CD8+ subsets (naïve, CM, EM, EMRA, RTE), CD4/CD8 ratio, total lymphocytes	MICT increased total lymphocytes and CD8+ EM T cells and maintained CD4+ naïve cells and recent thymic emigrants. HIIT decreased CD4+ T cells, naïve CD4+, RTE, and CD4/CD8 ratio. MICT produced anti-senescent, HIIT pro-senescent effects.	[170]
**RESISTANCE EXERCISE**						
**SET** (40% 1RM) vs. **IST** (80% 1RM) vs. passive stretching 3×/week *6 weeks*	Human RCT	N = 100 Women ≥65 years	Flow cytometry Dual-platform cell count	Senescence-prone T cells (CD28−/CD57+), Naïve T cells (CD28+/CD57−)	SET reduced senescence-prone T cells by 44% (CD8+ pool) and 51% (CD8− pool). The effect was observed only in CMV-seropositive women. IST had no significant effect.	[176]
**RT** (60–85% 1RM) 3×/week *12 weeks*	Human RCT	N = 23 Obese post- menopausal women 65.6 ± 2.6 years	ELISA DXA 1RM strength test LPS challenge	CRP, leptin, TNF-α, IL-6, LPS-stimulated TNF-α and IL-10, muscle strength	RT reduced CRP (−33%), leptin (−18%), and TNF-α (−29%) and improved strength (+44%), with no changes in body composition. LPS-stimulated IL-10 increased (+20%), indicating enhanced innate immune responsiveness.	[178]
**COMBINED AEROBIC AND RESISTANCE EXERCISE**						
**Combined strength** **and SET** (60% 1RM + RPE 15/20) 2 sessions/week 60 min/session *12 weeks*	Human RCT	N = 96 Elderly 50–70 years	Flow cytometry, mass spectrometry	CD8+ EMRA T cells, KA, QA QA/KA ratio	CD8+ EMRA T cells remained stable in the exercise group, whereas they increased in the control group. Plasma KA increased in the exercise group and decreased in controls. Adding a dietary intervention further reduced QA.	[179]
**Low-dose combined** **resistance &** SET *6 weeks*	Human RCT	N = 40 Elderly 70.3 ± 5.0 years	Flow cytometry Luminex multiplex assay	CD4+/CD8+ ratio T-cell subsets naïve, CM, EM, T-EMRA, IL-6, IL-8 IL-10, VEGF	CD4+/CD8+ ratio increased. IL-6, IL-8, IL-10, and VEGF all decreased. Muscle strength improved. Short-term, low-threshold training was sufficient to attenuate markers of immune aging.	[174]
**Aquatic combined** **exercise** **(aerobic +** **resistance)** 2×/week 45 min/session *28 weeks*	Human RCT (4-arm)	N = 102 Community- dwelling elderly 71–74 years	ELISA, Cell count	TNF-α, IL-10, IL-1β, IL-1ra, TNF-α/IL-10 ratio, hematological parameters	TNF-α decreased across all exercise groups. In the interval training group, IL-10 increased, improving the TNF-α/IL-10 ratio. The combined group reduced the IL-1β/IL-1ra ratio. Hematological parameters improved across all exercise groups.	[181]

**Table 5:** Summary of evidence strength by lifestyle intervention category. *CALERIE: Comprehensive Assessment of Long-term Effects of Reducing Intake of Energy; CR: caloric restriction; CRP: C-reactive protein; EMRA/TEMRA: effector memory T cells re-expressing CD45RA (terminally differentiated); HDAC: histone deacetylase; IL: interleukin; MBSR: mindfulness-based stress reduction; MIND: Mediterranean-DASH intervention for neurodegenerative delay; PREDIMED: Prevención con Dieta Mediterránea; RCT: randomized controlled trial; TNF-α: tumor necrosis factor alpha; VITAL: VITamin D and Omega-3 Trial; VZV: varicella-zoster virus. Evidence grades: HIGH = multiple large RCTs with consistent immune or inflammatory endpoints; MODERATE–HIGH = multiple RCTs with consistent findings but smaller sample sizes or limited immune-specific data; MODERATE = RCTs or large prospective cohorts with moderate consistency; LOW–MODERATE = limited RCTs, heterogeneous protocols, or predominantly observational data.*

Intervention	References	Study Type	Sample Size	Evidence Grade	Consistency	Effect of Intervention
**Mediterranean** diet	Estruch et al., 2018 (PREDIMED) [131]	Human RCT	N = 7447	HIGH	High	Multiple large RCTs, 30% reduction in major cardiovascular events, robust reductions in CRP and IL-6 confirmed
**MIND** diet	Fei et al., 2025 [143]	Human prospective cohort	N = 3777	MODERATE	Moderate	Seven-year follow-up, high adherence associated with lower IL-1, IL-6, CRP, and neutrophil count, observational design, consistent with Mediterranean and DASH diet evidence
**CR**	Meydani et al., 2016 (CALERIE-2) [150]	Human RCT	N = 218	MODERATE	Moderate (conflicting for severe CR)	Benefits depend critically on the degree of restriction, age at initiation, and individual health status, risks of sarcopenia and nutritional deficiency in the frail elderly
**Vitamin D** supplementation	Hahn et al., 2022 (VITAL) [107]	Human RCT	N = 25,871	HIGH	High for autoimmunity; moderate for other outcomes	22% reduction in autoimmune disease incidence over 5 years, protective effect attenuated after trial cessation; immune effects beyond autoimmunity are less consistent
**Aerobic** **exercise**	Abd El-Kader & Al-Shreef, 2018 [169]	Human RCT	N = 80	MODERATE–HIGH	High for inflammatory markers	Multiple consistent RCTs in older adults show that aerobic training is superior to resistance for immune outcomes, intensity, and duration, requiring further standardization
**Combined** **aerobic** **+ resistance** **exercise**	Boßlau et al., 2023 [179]; Despeghel et al., 2021 [174]	Human RCT	N = 96	MODERATE–HIGH	High for T-cell and inflammatory outcomes	Strongest immunological profile among exercise modalities; attenuates CD8+ EMRA T-cell accumulation; redirects kynurenine pathway toward kynurenic acid
**MBSR**	Davidson et al., 2003 [197]; Pascoe et al., 2017 (meta-analysis) [198]	Human RCT/meta-analysis	N~2000 (meta)	MODERATE	Moderate	Effects on CRP and vaccination responses confirmed, epigenetic pathway (HDAC downregulation) documented, long-term immune data limited
**Vaccination** (VZV/shingles)	Kim & Crimmins, 2026 [226]	Human prospective cohort	N = 3884	MODERATE–HIGH	High for specific endpoints	Lower systemic inflammation and slower epigenetic and transcriptomic aging in vaccinated vs. unvaccinated adults ≥ 70 years, cross-sectional comparison, no longitudinal follow-up
**Cold exposure**	Buijze et al., 2016 [219]; El-Ansary et al., 2024 [221]	Human RCT	N = 3018 (Buijze); 60 (El-Ansary)	LOW–MODERATE	Low (heterogeneous protocols)	29% reduction in sickness absence (Buijze 2016, adults aged 18–65 years, no older adults), short-term protocols only, no immune endpoint data in older populations; cardiovascular risks in older individuals must be considered
**Yoga/mind–body** **practices**	Morgan et al., 2014 (meta-analysis) [205]; Yeun & Kim, 2021 [208]	Meta-analysis of RCTs	N~1500 (meta)	LOW–MODERATE	Low–moderate (inconsistent across trials)	Moderate reductions in CRP across studies, inconsistent effects on cytokines, and high heterogeneity in yoga type, population, and outcome measures

## Data Availability

No new data were created or analyzed in this study. Data sharing is not applicable to this article.
